# Structural analysis and molecular dynamics simulation studies of HIV-1 antisense protein predict its potential role in HIV replication and pathogenesis

**DOI:** 10.3389/fmicb.2023.1152206

**Published:** 2023-03-20

**Authors:** Balakumaran Sathiyamani, Evangeline Ann Daniel, Samdani Ansar, Bennett Henzeler Esakialraj, Sameer Hassan, Prasanna D. Revanasiddappa, Amrutha Keshavamurthy, Sujata Roy, Umashankar Vetrivel, Luke Elizabeth Hanna

**Affiliations:** ^1^Department of Virology and Biotechnology, National Institute for Research in Tuberculosis, Chennai, Tamil Nadu, India; ^2^University of Madras, Chennai, India; ^3^Center for Bioinformatics, Vision Research Foundation, Sankara Nethralaya, Chennai, Tamil Nadu, India; ^4^Department of Biosciences and Nutrition, Karolinska Institutet, Huddinge, Sweden; ^5^Department of Biotechnology, Siddaganga Institute of Technology, Tumakuru, Karnataka, India; ^6^Department of Biotechnology, Rajalakshmi Engineering College, Chennai, Tamil Nadu, India

**Keywords:** human immunodeficiency virus, antisense protein, modeling, molecular dynamics simulation, tertiary structure

## Abstract

The functional significance of the HIV-1 Antisense Protein (ASP) has been a paradox since its discovery. The expression of this protein in HIV-1-infected cells and its involvement in autophagy, transcriptional regulation, and viral latency have sporadically been reported in various studies. Yet, the definite role of this protein in HIV-1 infection remains unclear. Deciphering the 3D structure of HIV-1 ASP would throw light on its potential role in HIV lifecycle and host-virus interaction. Hence, using extensive molecular modeling and dynamics simulation for 200 ns, we predicted the plausible 3D-structures of ASP from two reference strains of HIV-1 namely, Indie-C1 (subtype-C) and NL4-3 (subtype-B) so as to derive its functional implication through structural domain analysis. In spite of sequence and structural differences in subtype B and C ASP, both structures appear to share common domains like the Von Willebrand Factor Domain-A (VWFA), Integrin subunit alpha-X (ITGSX), and ETV6-Transcriptional repressor, thereby reiterating the potential role of HIV-1 ASP in transcriptional repression and autophagy, as reported in earlier studies. Gromos-based cluster analysis of the centroid structures also reassured the accuracy of the prediction. This is the first study to elucidate a highly plausible structure for HIV-1 ASP which could serve as a feeder for further experimental validation studies.

## 1. Introduction

A negative strand open reading frame (ORF) spanning the Human Immunodeficiency Virus-1 (HIV-1) genome, was identified in 1988, and termed as Antisense Protein (ASP; Miller, 1988; Dimonte, 2017). ASP has strong conservation among HIV-1 isolates, especially in group-M (Berger et al., 2015; Cassan et al., 2016; Liu et al., 2018), but is absent in HIV-2, SIV, and non-group M HIV-1 strains. Since its discovery, a number of attempts have been made to characterize the nature and function of HIV-1 ASP, but with limited success, as the protein has no known homologs and its function has continued to remain a source of debate. Not much is known about the structure of the protein either, except that it is ~189 amino acid long, hydrophobic, and has two transmembrane (TM) domains, two conserved cysteine triplets (CCC), and a predefined PxxPxxP motif (Savoret et al., 2020). The ASP ORF overlaps with the *env, rev*, and *tat* genes of HIV-1, and specific mutations in this gene have been linked to different gp120 tropic signatures, suggesting a possible association with viral tropism (Dimonte, 2017).

Ludwig et al. first reported the presence of a potential transcription initiator element situated in the antisense strand of the HIV-1 genome that generated antisense transcripts from the Transactivation Response element (TAR) region in the 3′ HIV-1 LTR (Ludwig et al., 2006), using the protocol of Haist et al. for the detection of antigenomic RNA (Haist et al., 2015). Subsequent studies provided evidence for the expression of the protein in HIV-1-infected cells and demonstrated its localization on the plasma membrane (Briquet and Vaquero, 2002; Clerc et al., 2011; Torresilla et al., 2013; Affram et al., 2019). The antibodies used for detection of HIV-1 ASP were raised against ASP residues 47–61 (Torresilla et al., 2013) and residues 97–110 in mice (Affram et al., 2019). Antibodies against ASP were first detected in HIV-1 patients’ sera as early as 1995 (Vanhée-Brossollet et al., 1995). More recently, cytotoxic CD8+ T-cell responses directed against various domains of ASP have been detected in HIV-1 patients, confirming ASP expression during HIV infection (Bet et al., 2015; Savoret et al., 2020).

Although there is emerging evidence to suggest that ASP has a role in HIV life cycle and pathogenesis, strong experimental evidence to support its cellular function is lacking. The present study was aimed to model the stable tertiary structure of ASP by implementing extensive molecular dynamics simulation and use it to predict the potential functional role of this protein in HIV-1 infection and pathogenesis. We modeled the ASP of two HIV-1 group M viruses, Indie-C1 (subtype C) and NL4-3 (subtype B), using I-TASSER and stringently constrained the secondary structural elements using MODELLER (V.10.1). The modeled structures were further iteratively refined until a high plausibility was achieved. Subsequently, DALI (Domain Alignment) structural alignment based of the predicted structures was performed with all the proteins in Protein Data Bank to predict the overlapping structural domains, and suggest the probable functions of this unique protein.

## 2. Methods

### 2.1. Retrieval of ASP open reading frame

Due to the high degree of variability in HIV-1 sequences, we restricted our analysis to the ASP of two well-characterized HIV-1 reference strains, namely, Indie-C1 (subtype C) and NL4-3 (subtype B strain). The full-length sequences of these viruses were downloaded from HIV Los Alamos database.^1^ A customized PERL program was scripted to generate the reverse complement of the retrieved sequences, followed by prediction of long ORFs (100 bp or longer) using “ATGCCC” as the start string and “TAA,” “TAG,” and “TGA” as the stop string. The retrieved ORFs were translated into amino acids using the ExPASy (Expert Protein Analysis System) translate tool (https://web.expasy.org/translate/; Gasteiger et al., 2003). Clustal Omega (ClustalO; https://www.ebi.ac.uk/Tools/msa/clustalo/; Madeira et al., 2022) was used to align the amino acid sequences to check for sequence conservation. Since the ASP ORF overlaps with the envelope ORF, we scanned the amino acid sequence of ASP for disordered regions/residues using the DISOPRED server (http://bioinf.cs.ucl.ac.uk/psipred/?disopred=1; Jones and Cozzetto, 2015).

### 2.2. Structure modeling and refinement

In order to identify a suitable structural template for homology modeling of HIV-1 ASP, BlastP search (Altschul et al., 1990) was done against the PDB database (Berman et al., 2000). Since no suitable template was identified, the ASP structure was modeled using the threading method with I-TASSER server (Zhang, 2008; Roy et al., 2010; Yang et al., 2015). Using the PSIPRED server, sequence-based secondary prediction was performed (McGuffin et al., 2000). The predicted structures were refined iteratively for proper assignment of secondary structure elements (SSE) through the constrained assignment module of Modeller (v.10.1; Martí-Renom et al., 2000). The resulting model was loop refined and geometry optimized using Modeller and Modrefiner (Xu and Zhang, 2011), respectively. Homology modeling was performed using Modeller software (v.10.1) by generating 50 models, and the least DOPE score (Shen and Sali, 2006) model was chosen for MD simulation for 200 ns using Desmond package (Bowers et al., 2006).

### 2.3. Molecular dynamics simulation of the refined structures

The modeled structures of ASP_Indie-C1 and ASP_NL4-3 were further geometry optimized using the academic version of Maestro (Schrödinger Release 2022-3: Maestro, 2021). Bond orders were assigned, and the structures were protonated using the PROPKA module (Madhavi Sastry et al., 2013) with pH set at 7.0. Subsequently, the structures were energy minimized using OPLS2005 force field (Shivakumar et al., 2012) with a Root Mean Square Deviation (RMSD) convergence cut-off of 0.3 Ǻ. The geometry optimized structures were subjected to MD simulation using Desmond-v4.5 with OPLS-2005 set as force field. The proteins were solvated in a cubical box using the SPC water model. The system was further neutralized by adding counter ions and energy minimized with a maximum iteration of 2,000 and 1.0 kcal/mol/Ǻ convergence threshold using the steepest descent and Limited-memory Broyden-Fletcher-Gold Farb-Shanno (LBFGS) algorithm (Liu and Nocedal, 1989). The bond length and bond angles of the molecules and geometry of water molecules were restrained using the SHAKE algorithm (Kräutler et al., 2001). Appropriate periodic boundary conditions were implemented and Particle Mesh Ewald was applied for long-range electrostatic calculations (Bulatov et al., 2011). The system was equilibrated in an NPT ensemble with a temperature of 300 K and pressure of 1 bar using Martyna-Tobias-Klein (Martyna et al., 1994) pressure coupling with the Nose-Hoover coupling algorithm (Nosé, 1984; Hoover, 1985). The well-equilibrated systems were further subjected to production runs for 200 ns. Subsequently, the Root Mean Square Deviation (RMSD), Root Mean Square Fluctuation (RMSF), Radius of Gyration, and secondary structure of MD trajectories were analyzed.

### 2.4. Cluster analysis of the molecular dynamics simulation trajectories

In order to determine the conformational sampling of both ASP-Indie-C1 and ASP-NL4-3 during the simulation, a cluster analysis was performed. For this, the Desmond trajectory files were converted for gromacs compatibility using Visual Molecular Dynamics (VMD; http://www.ks.uiuc.edu/Research/vmd/; Humphrey et al., 1996). The gromacs compatible trajectories were used as backbone for RMSD-based clustering using the g_cluster module of gromacs by implementing gromos method (Daura et al., 1999).

### 2.5. Prediction of structural homologs

The final frame conformation of both the models obtained from the MD simulation was subjected to structural comparison with all the experimentally determined structures in PDB using the DALI server (http://ekhidna2.biocenter.helsinki.fi/dali/; Holm and Rosenström, 2010). The top-scoring PDB structures based on Z-score (Holm et al., 2008) in terms of structural homology were further probed for overlapping domains in order to assign probable functional significance to the modeled structures.

## 3. Results

### 3.1. Retrieval of ASP ORF and amino acid sequence

**Figure 1:**
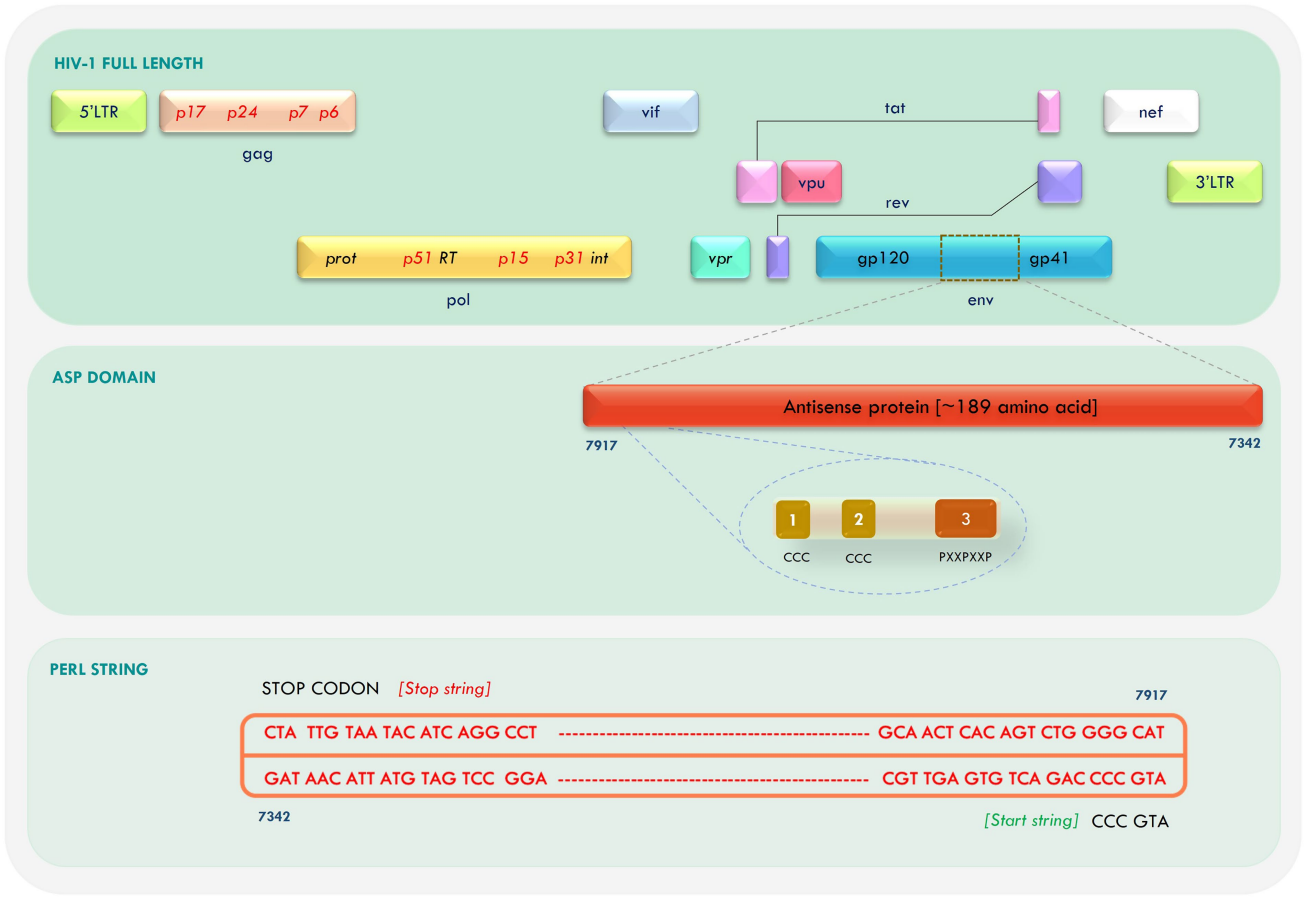
Mapping of antisense protein (ASP) in the Human Immunodeficiency Virus-1 (HIV-1) genome and retrieval of open reading frame (ORF).

The antisense ORF was retrieved from the Indie-C1 and NL4-3 whole-genome sequence using an in-house PERL script with “ATGCCC” as start string and the three stop codons as stop string (Figure 1). The ORFs were translated and Clustal Omega was used to align the sequences. A high degree of similarity was observed in the first 100 amino acid residues between the two sequences. We identified confined repeats of two cysteine triplets (CCC) followed by a (PxxPxxP) motif within the first 60 residues. The overall identity between the two sequences was 75.4%; the conservation gradually diminished toward the C-terminus.

### 3.2. Secondary structure prediction

**Figure 2:**
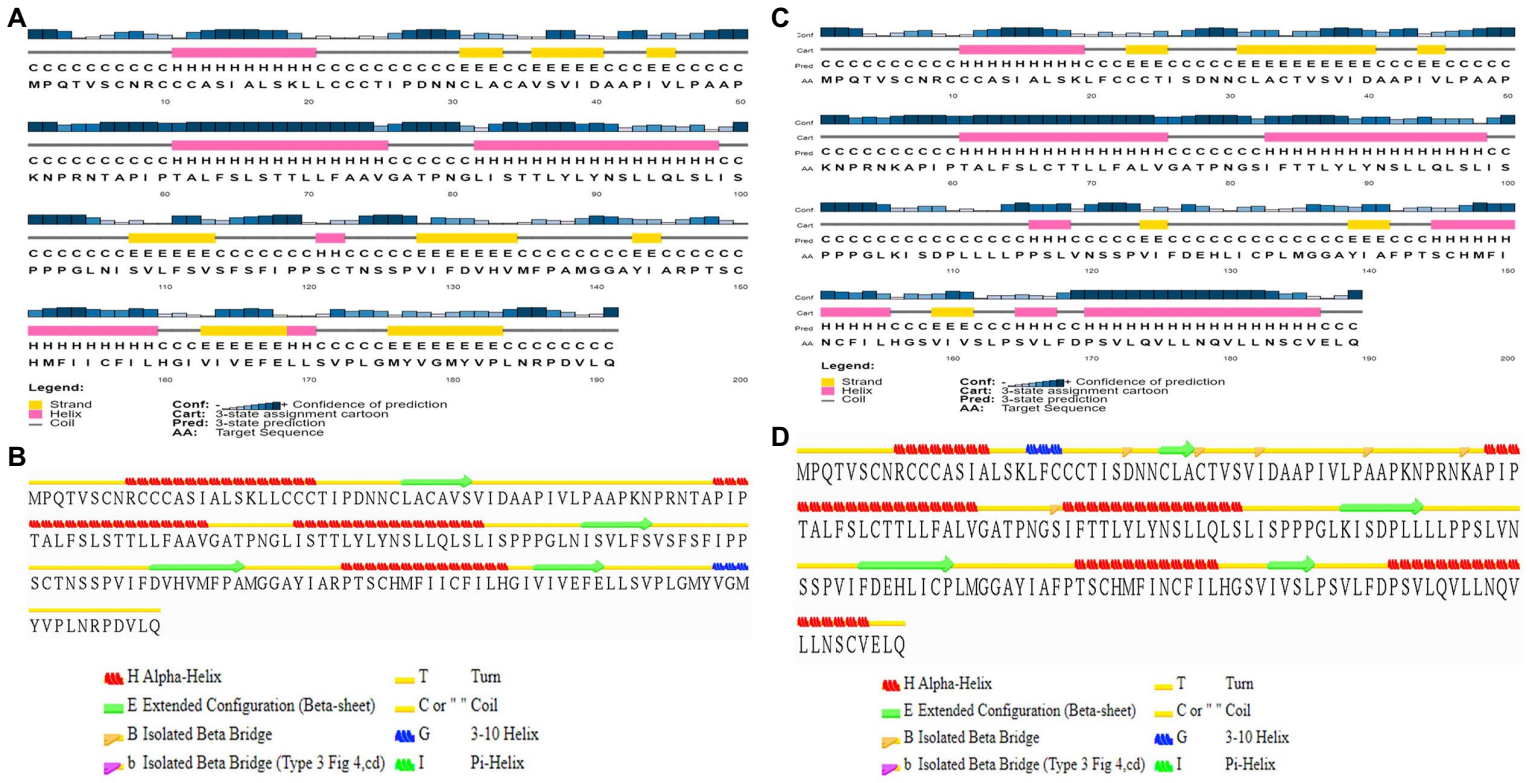
Sequence-based secondary structure prediction. **(A)** Sequence-based secondary structure assignment for ASP_Indie-C1 using PSI-PRED; **(B)** Secondary structure of the modeled ASP_Indie-C1 predicted using STRIDE; **(C)** Sequence-based secondary structure assignment for ASP_NL4-3 using PSIPRED; and **(D)** Secondary structure of the modeled ASP_NL4-3 predicted using STRIDE.

Sequence-based secondary structure prediction was attempted for the Indie-C1 and NL4-3 ASP (Figure 2) using PSIPRED 4 server (http://bioinf.cs.ucl.ac.uk/psipred/; McGuffin et al., 2000), and compared with the secondary structure elements in the 3D structure derived using STRIDE (http://webclu.bio.wzw.tum.de/cgi-bin/stride/stridecgi.py; Heinig and Frishman, 2004). Sequence-based secondary structure prediction was constrained in the predicted models. Non-homology of the antisense protein to any single PDB structure led us to implement the constrained strategy, as PSIPRED 4 has 84.2% prediction accuracy (Buchan and Jones, 2019). The secondary structures predicted by the two servers had discrepancies at the C-terminus. This difference can be attributed to the 25% dissimilarity found across the amino acid sequence when aligned with Clustal O (Supplementary Figure 1) and high dissimilarity found in the C-terminus region. The amino acid sequences were analyzed using DISOPRED to identify residues/regions contributing to structural disorder. It was found that only the terminal residues [Residue number—1 (in both NL4-3 and Indie-C1) and 191 (in Indie-C1)] contributed to some structural disorder, which was very negligible (Supplementary Figures 2A,B).

### 3.3. Molecular modeling of ASP_Indie-C1

**Figure 3:**
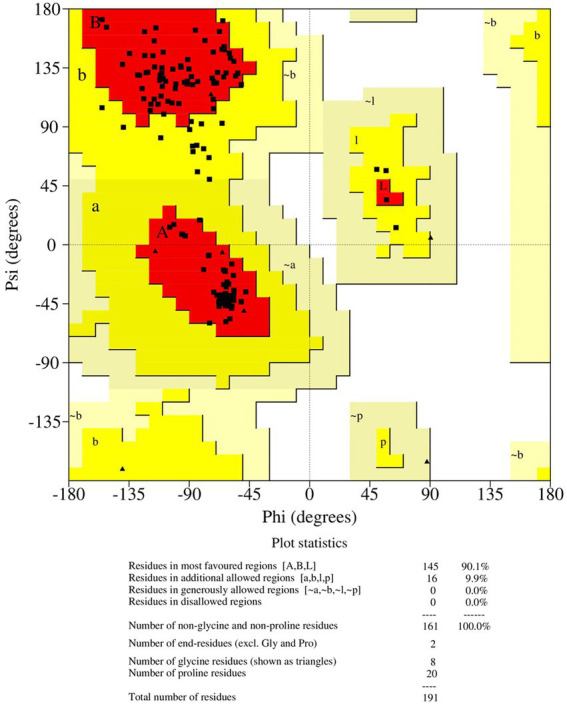
Ramachandran plot of the loop refined protein structure of ASP_Indie-C1.

Antisense protein_Indie-C1 sequence was submitted to I-TASSER server for molecular modeling. The modeled structure gave a significant C-score of −3.46. The structural compliance of sequence-based secondary structure elements (SSEs) in the predicted model was checked and proper SSEs were assigned using the restrained modeling module of MODELLER (v.10.1). The conformation with the least DOPE score (−20331.8) was proceeded for refinement using Modrefiner. The quality of the refined model was checked using the Ramachandran plot (Figure 3), and outliers were refined by sequential refinement and loop modeling. The final loop refined structure showed that 90% of the residues were in the most favored region with no residues in the disallowed or generously allowed regions. The structure also showed an ERRAT score of 68.9 (Supplementary Figure 4) and a PROSA score of −3.79 (Supplementary Figures 3A–C), indicating the good quality of the modeled structure.

### 3.4. Molecular dynamics simulation of ASP_Indie-C1

**Figure 4:**
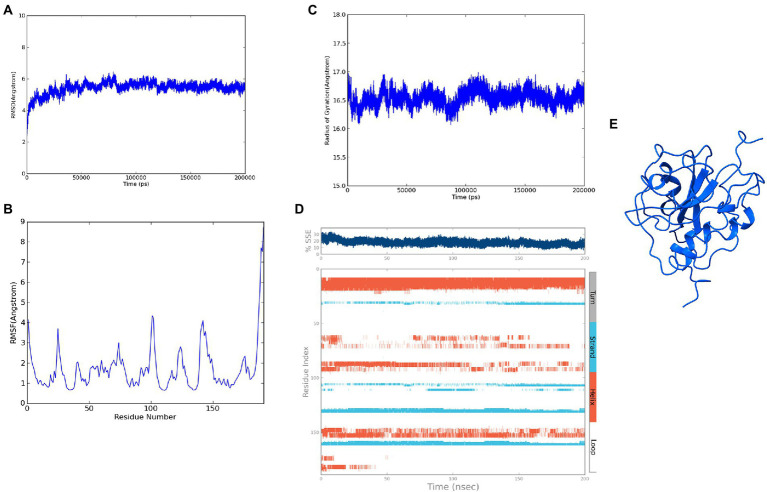
Molecular dynamics simulation trajectory analysis of ASP_Indie-C1: **(A)** Root Mean Square Deviation plot of ASP_Indie-C1 during 200 ns simulation period. **(B)** Root Mean Square Fluctuation plot of ASP_Indie-C1 during 200 ns simulation period. **(C)** Radius of gyration plot of ASP_Indie-C1 during 200 ns simulation period. **(D)** Secondary structure elements of ASP_Indie-C1 during simulation period (Orange-helices; Blue-strands; Gray-turns; White-loops). **(E)** Final frame structure of ASP_Indie-C1 after 200 ns simulation.

The modeled ASP_Indie-C1 structure was subjected to molecular dynamics simulation for 200 ns, and the resulting trajectory was analyzed for various parameters. The RMSD graph showed that the backbone was deviated up to 6Ǻ and deflected to ~5.6Ǻ from 80 ns onward and maintained throughout the simulation (Figure 4A). The RMSF plot showed a few residues in the N-terminus region to be fluctuating and reaching ~4.5 Ǻ, while the C-terminal residues showed fluctuation till ~9Ǻ (Figure 4B). The structural compactness during the simulation period was calculated using the radius of gyration plot (Figure 4C) and the structure was found to be highly compact as the gyration values were within ~1Ǻ. The protein secondary structure exhibited perturbations during the simulation period. It was observed that 24.68% of the residues formed the total secondary structure elements; 11.96% of these residues were in the helical form and 12.72% of the residues were present as strands during the simulation period (Figure 4D). The lowest potential energy conformation of −86110.98 kcal/mol was observed at 36.08 ns, while the final frame structure at the 200^th^ns showed a potential energy of −85521.29 kcal/mol (Figure 4E).

### 3.5. Molecular modeling of ASP_NL4-3

**Figure 5:**
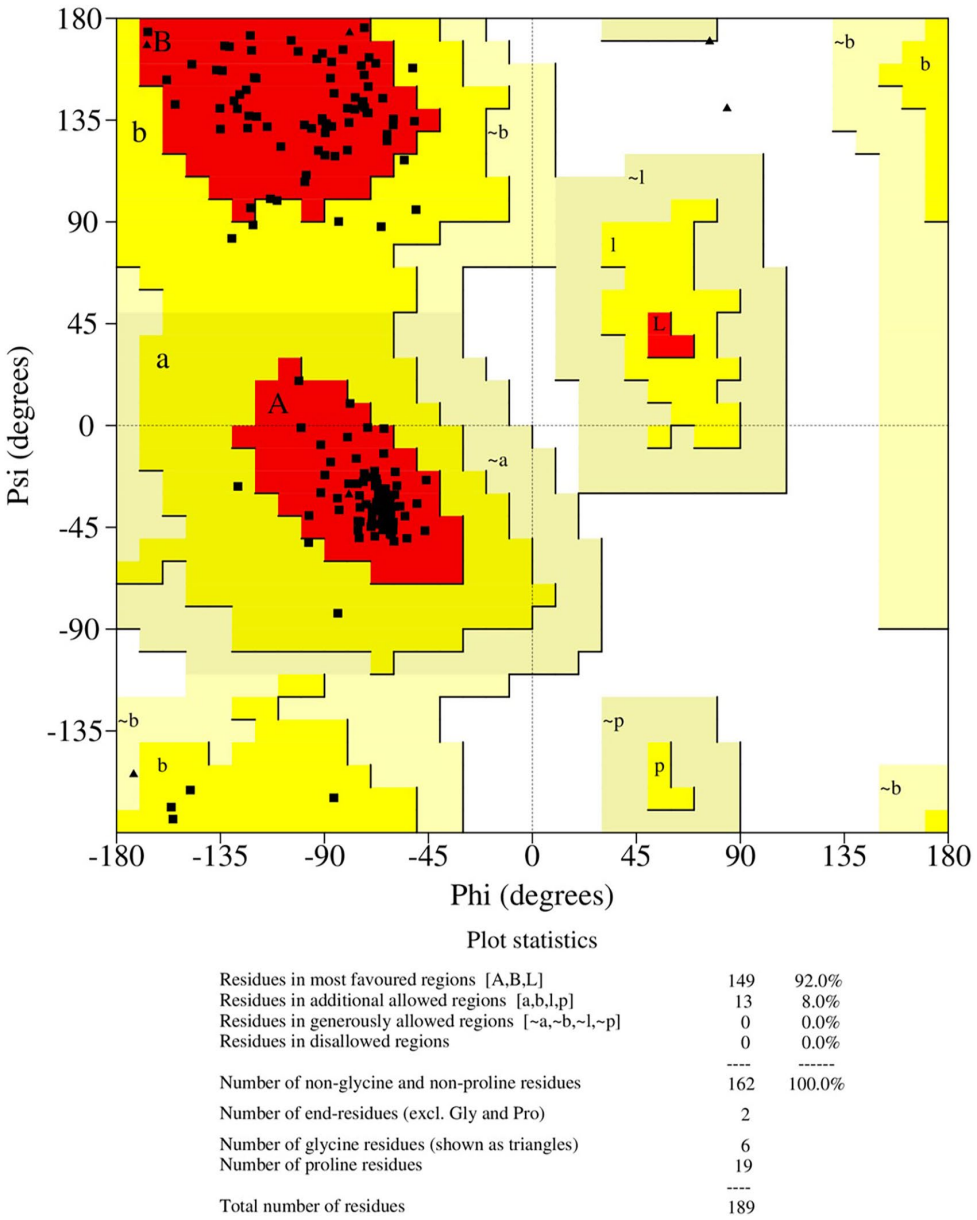
Ramachandran plot of the loop refined protein structure of ASP_NL4-3.

The final frame structure of ASP_Indie-C1 (Figure 4E) was chosen as the template to model the ASP_NL4-3 structure using Modeler (v.10.1), and the conformation with least DOPE score (−19670.75) was taken up for subsequent analysis. As in the case of ASP Indie-C1, the secondary structure elements of ASP_NL4-3 were used to constrain its 3D model so as to closely comply with the PSIPRED prediction (Figures 2C,D). The structure was further refined using Modrefiner. The quality of the refined structure was checked using the Ramachandran plot. Here, a few loop-forming residues were found in the disallowed region, and therefore, loop refinement was carried out. This resulted in 92% of the residues falling within the most favored region, and no residues in the disallowed or generously allowed regions (Figure 5). This structure gave an ERRAT score of 57.78 (Supplementary Figure 6) and PROSA score of −3.78 (Supplementary Figures 5A–C), which endorses the high plausibility and quality of the modeled structure.

### 3.6. Molecular dynamics simulation of ASP_NL4-3

**Figure 6:**
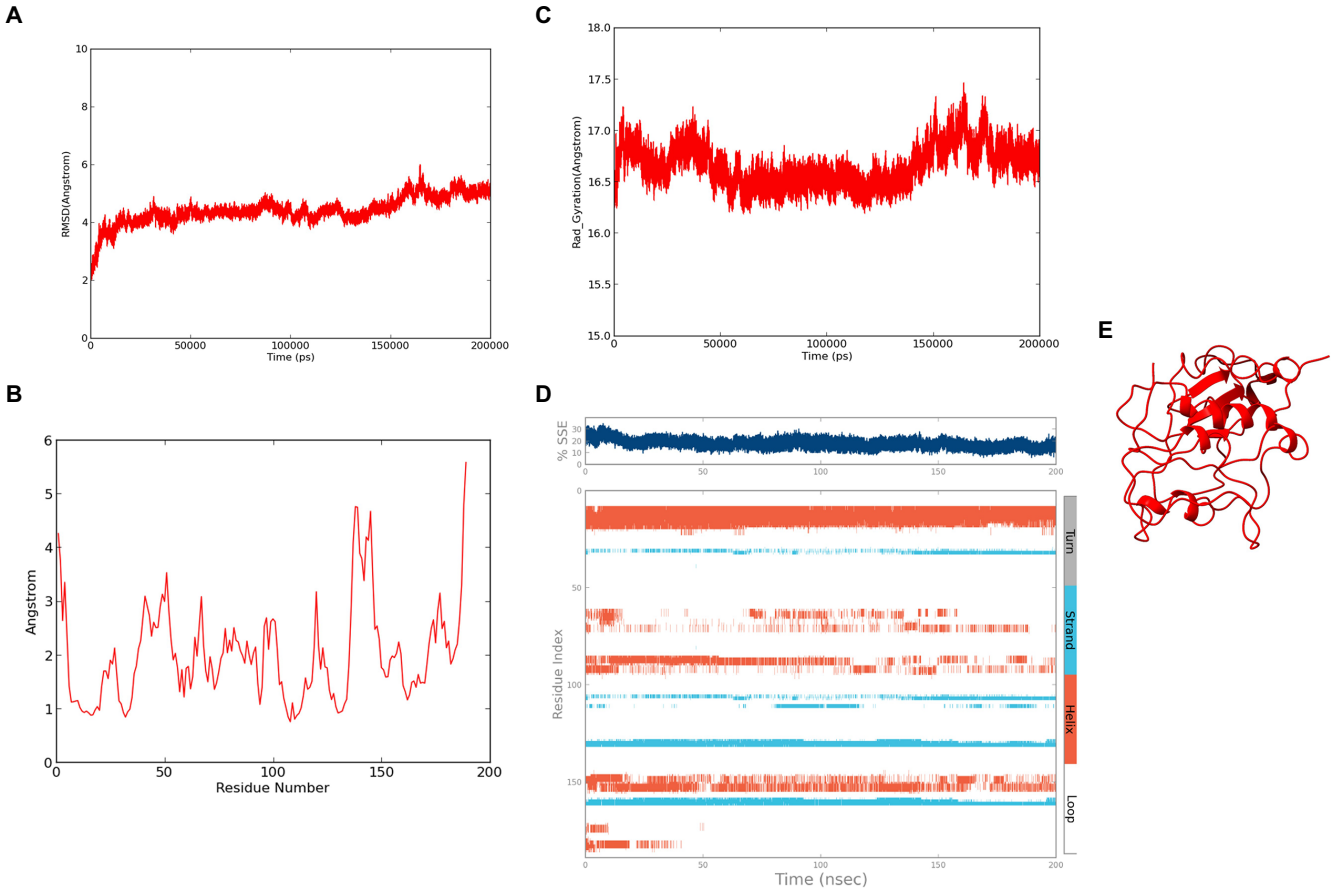
Molecular Dynamics simulation of ASP_NL4-3: **(A)** Root Mean Square Deviation plot of ASP_NL4-3 during the 200 ns simulation period. **(B)** Root Mean Square Fluctuation plot of ASP_NL4-3 during 200 ns simulation period. **(C)** Radius of gyration plot of ASP_NL4-3 during 200 ns simulation period. **(D)** Secondary structure elements of ASP_NL4-3 during simulation period (Orange-helices; Blue-strands; Gray-turns; White-loops). **(E)** Final frame structure of ASP_NL4-3 after 200 ns simulation.

The refined structure of ASP_NL4-3 was geometrically optimized using the Schrodinger Suite protein preparation wizard, and the model was dynamically simulated for 200 ns. From the RMSD plot, a deviation of 4Ǻ was observed in the initial period that stabilized around 180 ns with an RMSD of ~4.5Ǻ, and maintained throughout the simulation (Figure 6A). The RMSF plot showed that few residues in the N-terminus reached a fluctuation of ~4Ǻ, while the C-terminal residues showed fluctuation of upto ~6Ǻ (Figure 6B). The compactness of the protein structure during the simulation period was calculated using the radius of the gyration plot (Figure 6C). The gyration values were found to be within the range of ~1Ǻ, indicating the structural compactness of the modeled protein. Changes in the secondary structure of the protein during the simulation period were analyzed, and it was observed that 17.8% of residues formed the total secondary structure elements. The structure had 12.48% of the residues in the helix and 5.32% of the residues in the strand form throughout the MD simulation (Figure 6D). However, the secondary structure was found to be lost in a few regions during the simulation. The lowest potential energy of −99946.31 kcal/mol was observed at 74.78 ns, while the final frame structure at the 200th ns showed a potential energy of −99275.09 kcal/mol (Figure 6E).

### 3.7. Cluster analysis of the molecular dynamics simulation trajectories

**Figure 7:**
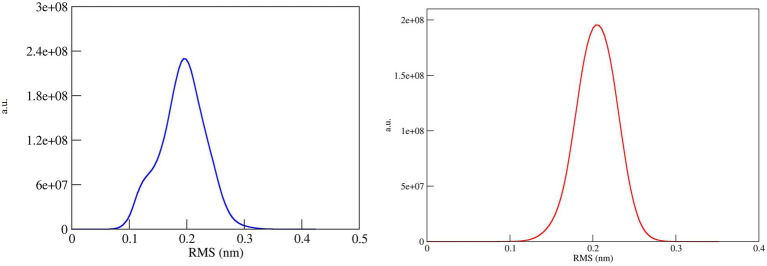
RMSD distribution plot for cluster analysis of ASP_Indie-C1 (Blue); ASP_NL4-3 (Red): Both the trajectories show a deviation of ~0.2 nm.

**Figure 8:**
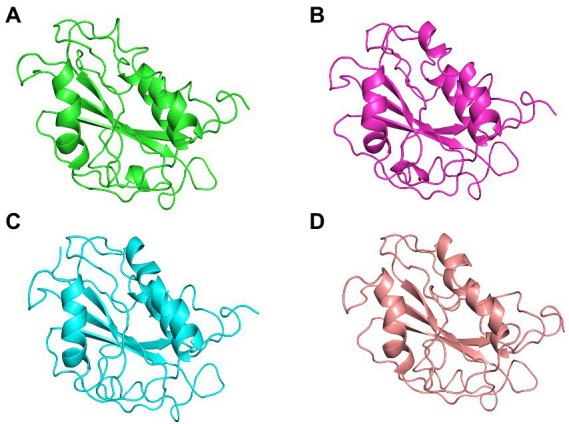
The centroid structures of each cluster attained from the clustering of ASP_Indie-C1 trajectory **(A)** Cluster 1 (C1) 1,000th ps, **(B)** Cluster 2 (C2) 81,100th ps, **(C)** Cluster 3 (C3) 29,800th ps, and **(D)** Cluster 4 (C4) 50,500th ps.

**Figure 9:**
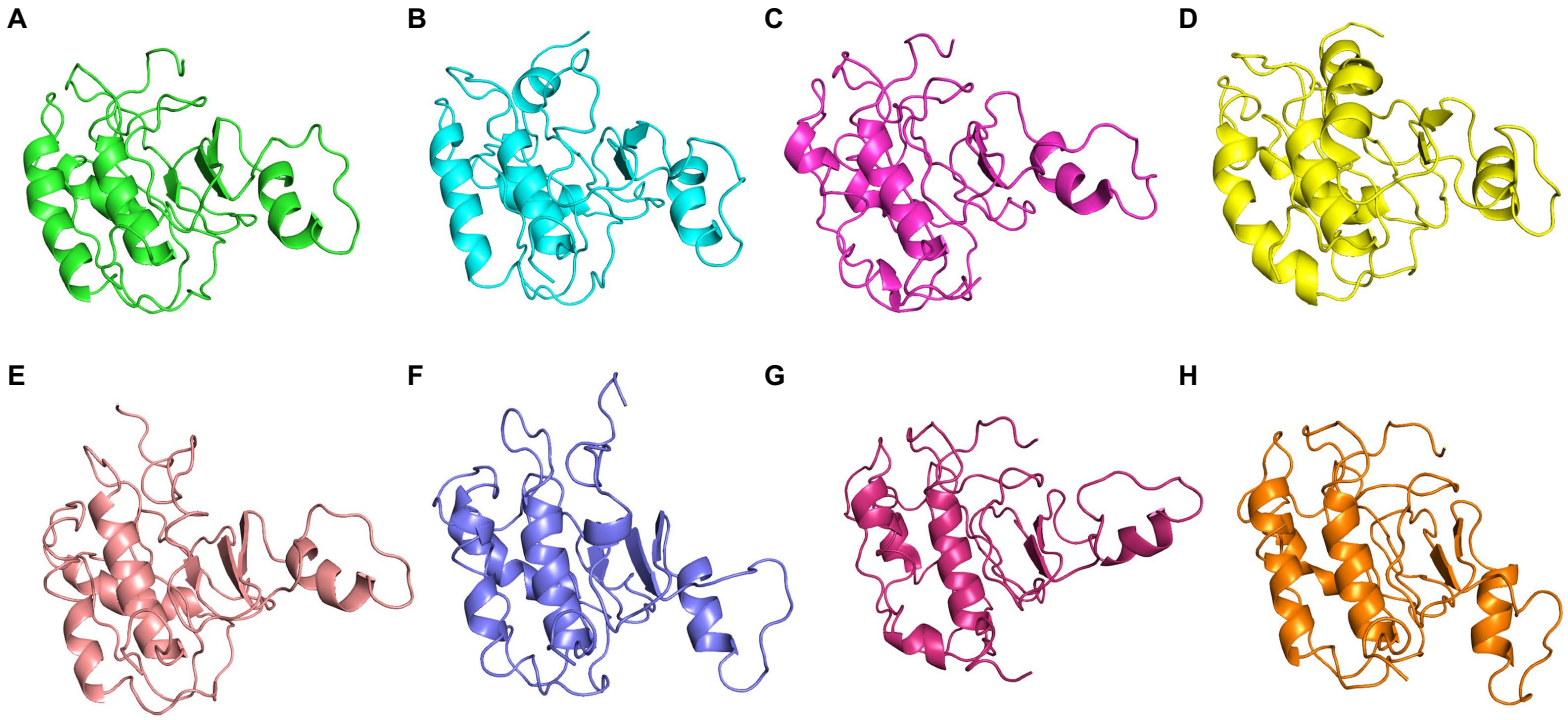
The centroid structure of each cluster obtained from the clustering of ASP_NL4-3 trajectory **(A)** Cluster 1 (C1)—5,940th ps, **(B)** Cluster 2 (C2) 11,600th ps, **(C)** Cluster 3 (C3) 13,640th ps, **(D)** Cluster 4 (C4) 1,200th ps, **(E)** Cluster 5 (C5) 33,500th ps, **(F)** Cluster 6 (C6) 13,750th ps, **(G)** Cluster 7 (C7) 31,500th ps, and **(H)** Cluster 8 (C8) 98,400th ps.

The conformational stability of both ASP_Indie-C1 and ASP_NL4-3 throughout the simulation was determined by cluster analysis. Based on the RMSD distribution, it was observed that both trajectories had a deviation of ~0.2 nm (Figure 7). Therefore, clustering was performed with a cutoff value of 0.2 nm for both trajectories based on gromos method. This resulted in four and eight prominent clusters for ASP-Indie-C1 and ASP-NL4-3, respectively. The representative centroid structure for each cluster was obtained at the timeframe as shown in Figures 8, 9 respectively. The average potential energy attained was −85451.782 ± 90.820 and − 80487.372 ± 118.227, respectively, by ASP_Indie-C1 and ASP_NL4-3. The structures with the least potential energy (at 55 and 75th ns, respectively, by ASP_Indie-C1 and ASP_NL4-3) were found to exhibit stable conformations (Supplementary Figures 7A–D).

### 3.8. Comparative analysis of structural homologs

**Figure 10:**
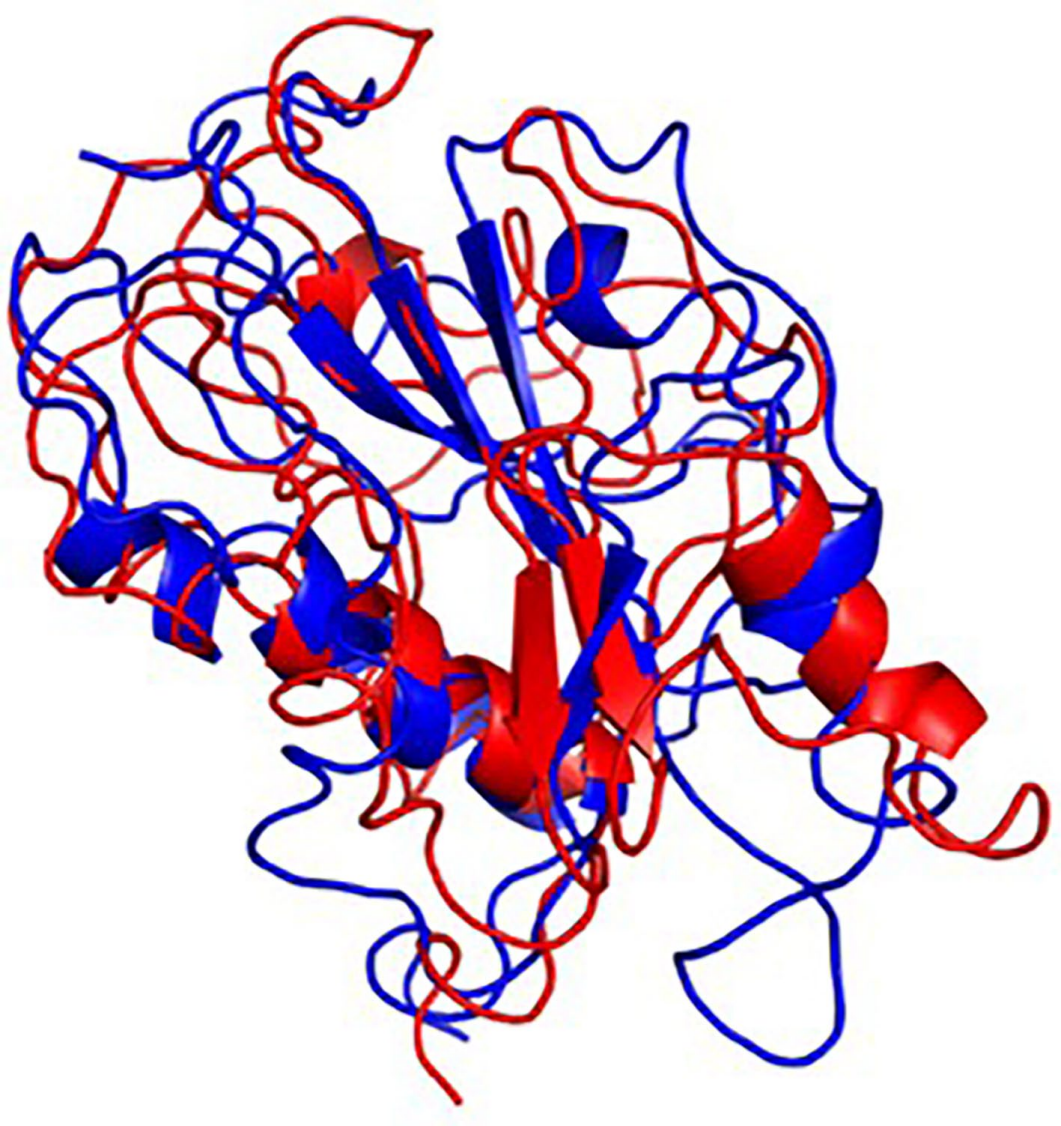
Final frame superimposition of the two models: Superimposed final frame of ASP_Indie-C1 (Blue) and ASP_NL4-3 (Red).

**Figure 11:**
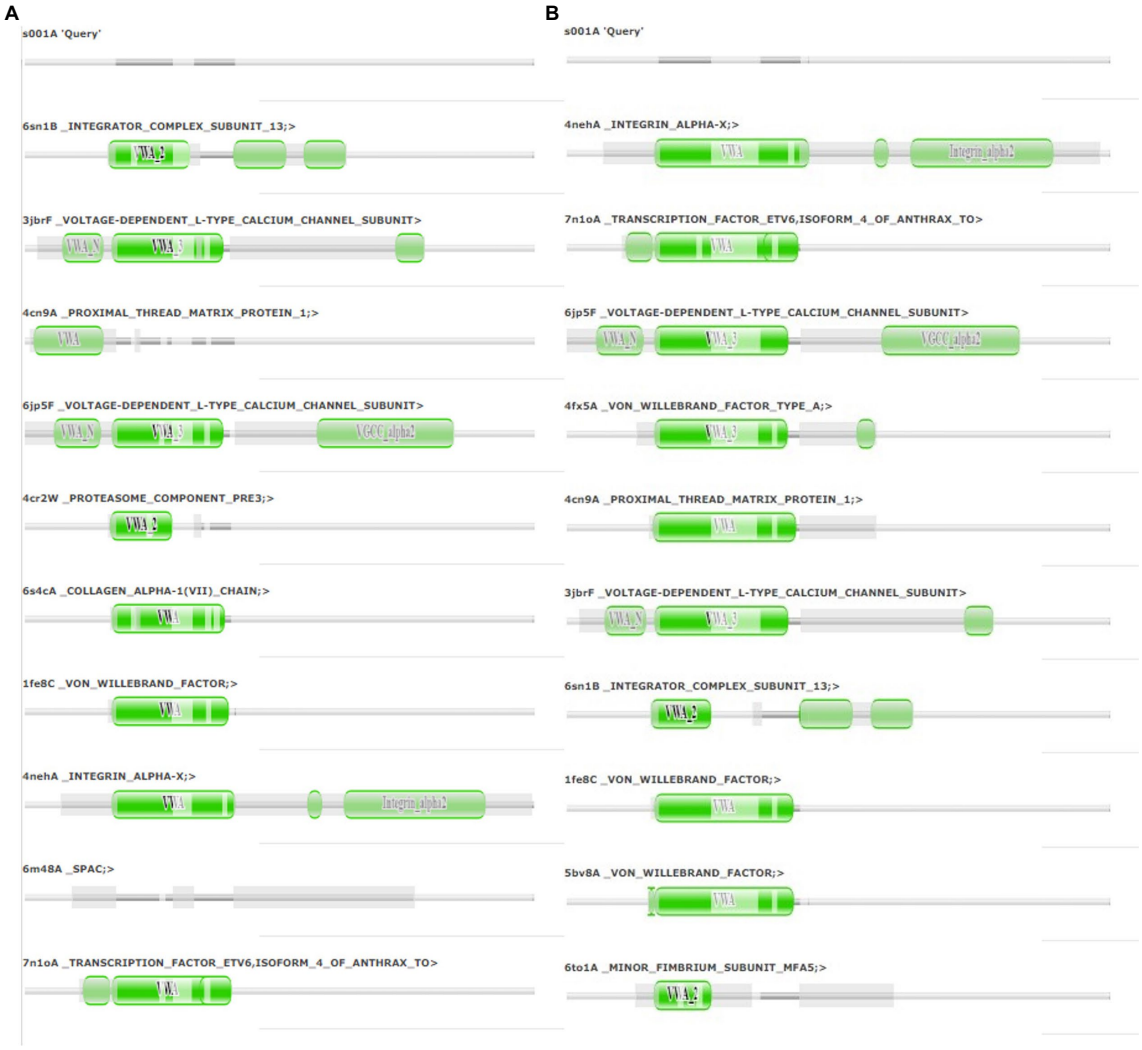
Pfam alignment from DALI server: **(A)** Alignment of ASP_NL4-3 with top 10 ranking matches from DALI. **(B)** Alignment of ASP_Indie-C1 with top 10 ranking matches from DALI. Dark green shading marks structural equivalence with the query structure, light green shading means the domain is located in an unaligned region. Structurally equivalent domain families are shown as vertically aligned dark green blobs.

The final frame structures of ASP_Indie-C1 and ASP_NL4-3 resulting from the MD trajectories were superimposed on each other (Figure 10) and compared. Subsequently, the final frame structures were submitted to DALI server for structural alignment with all the experimentally determined protein structures in PDB database. The top 10 hits for both the structures were compared and analyzed (Table 1; Figures 11A,B). The structures were found to have RMSD less than 4Ǻ and Z-score (significant similarity score) greater than 2. From the results, it was found that ASP_Indie-C1 and ASP_NL4-3 aligned with the VWFA domain (visualized using pfam in DALI) present in all the top-ranking protein structures. We also submitted a representative frame structure from each centroid cluster of both ASP_Indie-C1 and ASP_NL4-3 to the DALI server and obtained similar hits (Supplementary Figures 8, 9).

**Table 1:** List of common and unique hits from DALI for Indie-C1 and NL4-3.

Indie-C1					Common hits from DALI	NL4-3				
Z	rmsd	lali	nres	%ID	PDB description (chain)	Z	rmsd	lali	nres	%ID
6.3	3.8	139	1,083	4	Integrin alpha-x (4neh-A)	3.7	3.8	117	1,083	3
6.1	3.9	142	973	7	Voltage-dependent L-type calcium channel (6jp5-F)	4.1	3.9	121	973	4
6.1	3.5	132	257	8	Transcription factor ETV6, isoform 4 (7n1o-A)	3.7	3.6	115	257	7
5.7	4.1	140	872	7	Voltage-dependent L-type calcium channel (3jbr-F)	4.3	3.8	120	872	3
5.5	4.4	143	503	8	Integrator complex subunit 13 (6sn1-B)	4.5	4	124	503	12
5.4	3.6	132	397	6	Proximal thread matrix protein 1 (4cn9-A)	4.3	3.9	123	397	7
5.3	3.8	138	191	9	Von willebrand factor (1fe8-C)	3.8	3.7	119	191	8
					**Unique hits from DALI**					
5.9	3.8	136	426	8	Von willebrand factor type A (4fx5-A)					
5.3	4.1	143	209	8	Von willebrand factor (5bv8-A)					
5.3	3.8	137	566	8	Minor fimbrium subunit MFA5 (6to1-A)					
					Proteasome component PRE3 (4cr2-W)	4	3.8	110	197	6
					Collagen alpha-1(VII) Chain (6s4c-A)	3.9	3.7	115	185	7
					SPAC (6 m48-A)	3.7	4.6	136	813	9

Z, optimized similarity score; rmsd, root mean square deviation; lali, length of alignment; nres, total number of residues; and %ID, percent identity.

## 4. Discussion

The role of HIV-1 Antisense Protein continues to remain an enigma even after three decades of its initial discovery. Though several lines of evidence suggest possible roles for ASP in viral transcription/translation, autophagy, latency, viral release, and spread, the exact role of the protein in HIV life cycle and disease pathogenesis is unclear. Absence of a known homolog and lack of a three-dimensional structure are major challenges to defining the precise function of this protein. In the present study, we modeled a stable three-dimensional structure for the protein and used it to uncover clues regarding its potential role in HIV infection.

Due to high C-terminus variability in the ORFs of ASP, we could not derive a full-length consensus sequence for the protein. We therefore, used the ASP sequence of Indie-C1 and NL4-3, two well-characterized reference strains for HIV-1 subtype C and subtype B, respectively, for structure prediction. Using an in-house scripted PERL program, the ASP ORFs were retrieved from the antisense strand of the Indie-C1 and NL4-3 genome. While the C-terminus region showed a significant level of sequence variability, the N-terminus region was more or less well conserved. Both sequences had two conserved CCC- and SH3-binding motifs at the N-terminal region. This feature has been reported in earlier studies which analyzed the expression and localization of ASP in NL4-3-infected Jurkat cells. Overlapping ORFs could contain residues that favor structural disorderliness in proteins resulting in requirement of a binding partner for stabilization (Pavesi et al., 2018). Hence, we analyzed the ASP sequence for the presence of potential residues that might contribute to structural disorder using the DISOPRED server but did not find any significant core residues contributing to structural disorderliness. Secondary structure prediction by PSIPRED and STRIDE identified two major transmembrane (TM) helices in Indie-C1 and NL4-3 ASP. Clerc et al. also demonstrated sub-cellular localization of the protein using the wild type and mutant ASP, ASPmut66, which lacked the transmembrane domain. The mutant protein did not localize on the plasma membrane, and showed a different staining pattern as compared to the wild-type protein (Clerc et al., 2011), providing experimental proof to demonstrate the presence of TM domains in ASP and suggesting its prominent association with the plasma membrane.

We first modeled the tertiary structure of ASP using the Indie-C1 sequence, and subsequently used it as the template for modeling the ASP_NL4-3 structure. The best model of ASP_Indie-C1 gave a significant C-score of −3.46 in I-Tasser. We also used AlphaFold2 (https://colab.research.google.com/github/sokrypton/ColabFold/blob/main/AlphaFold2.ipynb; Jumper et al., 2021) to predict the 3D structure (data not shown), but the model did not conform to the secondary structure elements predicted by PSIPRED and STRIDE; hence, we chose the model predicted by I-Tasser which had higher order of agreement with SSE prediction. This model was refined to assign appropriate secondary structure elements using Modeller (v.10.1). The least DOPE score model was refined using Modrefiner. The quality of the refined structure was evaluated using Ramachandran plot, PROSA, and SAVES. The final model was prepared using the protein preparation wizard of the Schrodinger Suite, and Molecular Dynamics simulation (MD) was carried out using Desmond for a time scale of 200 ns. As seen from the RMSD plot of ASP_Indie-C1, deviations were observed till the simulation reached 80 ns, but subsequently achieved stability. A gradual increase in fluctuation in RMSF was observed at the carboxyl end indicating inconsistency at the C-terminus of the protein. The protein exhibited compactness after 80 ns of simulation. The Rg was found to be ~16.7Ǻ from the radius of gyration graph. We observed four helices (~10–30, ~60–70, ~80–95, and ~150–160) and four strands (~35–40, ~105–110, ~130–135, and ~160–163) in the SSE plot which correlates with the predicted secondary structure (Figure 2A). Taking into account all the above parameters, we postulate that the final frame structure at 200 ns could represent a stable structure for ASP.

The tertiary structure of ASP_NL4-3 was generated using ASP_Indie-C1 as the template and the structure was modeled using Modeller (v.10.1). The model with the lowest DOPE score was further refined and the model quality was assessed using Ramachandran plot, PROSA, and SAVES. The RMSD and Rg plot indicate that the ASP_NL4-3 structure attained stability only at the end of the simulation period of 180 and 160 ns, respectively, with an Rg value of ~16.7Ǻ. Though RMSF showed high fluctuation, SSE analysis revealed the presence of two helices at the carboxyl end, of which one was diminished after 30 ns of simulation. The ASP_NL4-3 structure also had four helices and strands like the ASP_Indie-C1 structure. Though the minor strands predicted by PSIPRED were not observed, the major helices and strands were present in both the predicted secondary structures. The final frame models of both ASPs were superimposed to check for structural resemblance, which inferred a backbone RMSD of 6.637Ǻ. Though the ~75% sequence similarity was found between the two ASPs the models feature large conformational evolution during MD simulation, wherein the conformational differences were due to the lower sequence homology at the C-terminus. The final frames were submitted to DALI server for structural comparison with the PDB entries. The top 10 hits were selected for each model, and the common hits were identified (Table 1). The functional significance of the identified homologs was analyzed to predict the probable biological role of ASP in HIV infection.

One of the shared structures predicted in the topmost hits was the Von Willebrand Factor domain-A (VWFA), which is present across the family of integrins, complement factors, collagens, and numerous other extracellular proteins.^2^ These proteins function as multimeric protein complexes and play a role in key biological events such as cell–cell adhesion, cell migration, and signal transduction. We found that around 40% of the amino acid residues across ASP aligned with the VWA domain, suggesting that this domain could likely be conformational rather than linear (Supplementary Figures 10A,B). ASP was also predicted to share homology with ETV6 which is a transcriptional repressor. A histone acetyl transferase protein called TIP60 serves as co-repressor along with ETV6 (Nordentoft and Jørgensen, 2003). An earlier study showed that HIV-1 tat interacts with TIP60 leading to inhibition of its acetyl transferase activity (Creaven et al., 1999). Col et al. also reported that tat-mediated TIP60 inhibition affects important regulatory events such as DNA repair and apoptosis due to DNA damage. Tat intercepts TIP60-facilitated apoptosis and provides sufficient time for proviral DNA transcription to increase virion production (Col et al., 2005). HIV-1 antisense transcription was found to be more active in monocyte-derived cells than in activated T cells, and unaltered in the absence of tat (Laverdure et al., 2012). Tat-mediated downregulation of ASP has also been reported (Michael et al., 1994), and this negative correlation is thought to be due to 5′ and 3′ LTR transcription.

The Integrator Complex Subunit 13 (INTS13) was another hit predicted by DALI. Very interestingly, using HIV-1 promoter and genome wide analysis, Stadelmayer et al. showed that the Integrator Complex subunit’s (INSTcom) target genes were enriched in the HIV-1 transactivation response (TAR) element/negative elongation factor (NELF)-binding element. They demonstrated that RNAPII pause-release was mediated by NELF at/from coding genes that was controlled by INSTcom, thereby affecting their processivity (Stadelmayer et al., 2014). Here, we identified a link between HIV-1 ASP and INSTcom, indicating a possible role for the protein in transcriptional repression and gene regulation. Zapata et al. went on to show that the ASP RNA transcripts interact with and recruit polycomb repressor complex 2 (PRC2) to the HIV-1 5′ LTR, leading to reduced binding of RNAPII and mRNA processivity (Zapata et al., 2017). They hypothesize that epigenetic silencing of the HIV-1 5’LTR through the PRC2 pathway could contribute to proviral latency.

The Integrin subunit alpha-X (ITGSX) came up as another hit near the C-terminus of the protein in the DALI prediction. Although we observed a reduced number of residues in subtype B ASP as compared to subtype C particularly at the C-terminus, the two functional domains predicted at the C terminus of the protein, i.e., Voltage Gate Calcium Channels alpha 2 and Integrin alpha 2, were conserved in both the models. ITGSX is known to form a complex with β2 integrin (CD18) and aggregate in the intracellular compartment as well as the plasma membrane of monocyte-derived macrophages (MDM; Pelchen-Matthews et al., 2012). ASP was found to translocate to the cytoplasm from the nucleus and localize on the cell membrane when U1C8 cells were stimulated with PMA (Affram et al., 2019). This observation provides support to the probable membrane localization property of ASP and suggests that it could be an integral cell surface protein. Multiple hypotheses suggest that ASP has a direct or indirect involvement in HIV-1 replication by inducing autophagy in monocytes (Vanhée-Brossollet et al., 1995; Torresilla et al., 2013). ASP has also been reported to induce autophagy in monocytic cells through its interaction with the autophagy markers, LC3b-II and Beclin 1 (Torresilla et al., 2013). Another study also demonstrated autophagy inducing property of ASP in different clades of HIV-1 and its co-localization with p62 and LC3-II in autophagosome-like structures (Liu et al., 2018). Thus, it appears that ASP within the nucleus can cause transcriptional repression, and could also shuttle from the cytoplasm to localize on the plasma membrane, and induce autophagy by interacting with LC3b II and Beclin 1 for successful virion release. The presence of a cysteine-rich region in HIV-1 ASP suggests strong agglomeration/multimerization of the protein, which also links it with the autophagic pathway. In order to determine the consistency of domain occurrence across the MD trajectory, one representative centroid structure per gromos-based cluster was subjected to DALI analysis. This revealed that VWFA and ITGSX were consistently represented in all the centroid structures of the cluster, thereby indicating the predictive accuracy of the structural bioinformatics methods implemented.

## 5. Conclusion

This study is the first attempt to predict a plausible tertiary structure for HIV-1 ASP. Through in-depth bioinformatics analysis, we identified a number of structural homologs that provide valuable clues to the potential role of this protein in viral replication and disease pathogenesis. The most plausible functions of this protein as predicted from the hits appear to be transcriptional repression, autophagy, and viral latency. Prospective studies aimed at confirming the function of ASP in experimental studeis would provide deeper insights into its role in HIV infection, and unravel clues for potential therapeutic interventions to cure HIV-1 infection.

## Data availability statement

The original contributions presented in the study are included in the article/Supplementary material, further inquiries can be directed to the corresponding authors.

## Author contributions

LH, UV, and SH: conceptualization. LH, UV, SH, and BS: methodology. SA, PR, and AK: software. BS, ED, BE, AK, and SR: formal analysis. LH and UV: investigation. UV and SA: data curation. ED, BS, LH, and UV: writing—original draft preparation. SH, PR, SR, and BE: writing—review and editing. LH: supervision. All authors contributed to the article and approved the submitted version.

## Conflict of interest

The authors declare that the research was conducted in the absence of any commercial or financial relationships that could be construed as a potential conflict of interest.

## Publisher’s note

All claims expressed in this article are solely those of the authors and do not necessarily represent those of their affiliated organizations, or those of the publisher, the editors and the reviewers. Any product that may be evaluated in this article, or claim that may be made by its manufacturer, is not guaranteed or endorsed by the publisher.

## Footnotes

^1^ https://www.hiv.lanl.gov/content/index

^2^ https://www.ebi.ac.uk/interpro/entry/InterPro/IPR036465/

## References

[ref1] Affram Y. Zapata J. C. Gholizadeh Z. Tolbert W. D. Zhou W. Iglesias-Ussel M. D.. (2019). The HIV-1 antisense protein ASP is a transmembrane protein of the cell surface and an integral protein of the HIV-1 viral envelope. J. Virol. 93, 1–16. doi: 10.1128/jvi.00574-19, PMID: 31434734 PMC6803264

[ref2] Altschul S. F. Gish W. Miller W. Myers E. W. Lipman D. J. (1990). Basic local alignment search tool. J. Mol. Biol. 215, 403–410. doi: 10.1016/S0022-2836(05)80360-2 2231712

[ref3] Berger C. T. Llano A. Carlson J. M. Brumme Z. L. Brockman M. A. Cedeño S.. (2015). Immune screening identifies novel T cell targets encoded by antisense Reading frames of HIV-1. J. Virol. 89, 4015–4019. doi: 10.1128/jvi.03435-14, PMID: 25589651 PMC4403399

[ref4] Berman H. M. Westbrook J. Feng Z. Gilliland G. Bhat T. N. Weissig H.. (2000). The protein data bank. Nucleic Acids Res. 28, 235–242. doi: 10.1093/nar/28.1.235, PMID: 10592235 PMC102472

[ref5] Bet A. Maze E. A. Bansal A. Sterrett S. Gross A. Graff-Dubois S.. (2015). The HIV-1 antisense protein (ASP) induces CD8 T cell responses during chronic infection. Retrovirology 12:15. doi: 10.1186/s12977-015-0135-y, PMID: 25809376 PMC4335690

[ref6] Bowers K. J. Chow D. E. Xu H. Dror R. O. Eastwood M. P. Gregersen B. A.. (2006). “Scalable algorithms for molecular dynamics simulations on commodity clusters.” in *SC’06: Proceedings of the 2006 ACM/IEEE conference on supercomputing*. 43.

[ref7] Briquet S. Vaquero C. (2002). Immunolocalization studies of an antisense protein in HIV-1-infected cells and viral particles. Virology 292, 177–184. doi: 10.1006/viro.2001.1224, PMID: 11878921

[ref8] Buchan D. W. A. Jones D. T. (2019). The PSIPRED protein analysis workbench: 20 years on. Nucleic Acids Res. 47, W402–W407. doi: 10.1093/nar/gkz297, PMID: 31251384 PMC6602445

[ref9] Bulatov V. V. Rhee M. Cai W. (2011). Periodic boundary conditions for dislocation dynamics simulations in three dimensions. MRS Online Proc. Libr. 653:13. doi: 10.1557/PROC-653-Z1.3

[ref10] Cassan E. Arigon-Chifolleau A.-M. Mesnard J.-M. Gross A. Gascuel O. (2016). Concomitant emergence of the antisense protein gene of HIV-1 and of the pandemic. Proc. Natl. Acad. Sci. 113, 11537–11542. doi: 10.1073/pnas.1605739113, PMID: 27681623 PMC5068275

[ref11] Clerc I. Laverdure S. Torresilla C. Landry S. Borel S. Vargas A.. (2011). Polarized expression of the membrane ASP protein derived from HIV-1 antisense transcription in T cells. Retrovirology 8:74. doi: 10.1186/1742-4690-8-74, PMID: 21929758 PMC3182985

[ref12] Col E. Caron C. Chable-Bessia C. Legube G. Gazzeri S. Komatsu Y.. (2005). HIV-1 tat targets Tip60 to impair the apoptotic cell response to genotoxic stresses. EMBO J. 24, 2634–2645. doi: 10.1038/sj.emboj.7600734, PMID: 16001085 PMC1176461

[ref13] Creaven M. Hans F. Mutskov V. Col E. Caron C. Dimitrov S.. (1999). Control of the histone-Acetyltransferase activity of Tip60 by the HIV-1 Transactivator protein, tat. Biochemistry 38, 8826–8830. doi: 10.1021/bi9907274, PMID: 10393559

[ref14] Daura X. Gademann K. Jaun B. Seebach D. van Gunsteren W. F. Mark A. E. (1999). Peptide folding: when simulation meets experiment. Angew. Chem. Int. Ed. 38, 236–240. doi: 10.1002/(SICI)1521-3773(19990115)38:1/2<236::AID-ANIE236>3.0.CO;2-M

[ref15] Dimonte S. (2017). Different HIV-1 env frames: gp120 and ASP (antisense protein) biosynthesis, and theirs co-variation tropic amino acid signatures in X4-and R5-viruses. J. Med. Virol. 89, 112–122. doi: 10.1002/jmv.24611, PMID: 27328810

[ref16] Gasteiger E. Gattiker A. Hoogland C. Ivanyi I. Appel R. D. Bairoch A. (2003). ExPASy: the proteomics server for in-depth protein knowledge and analysis. Nucleic Acids Res. 31, 3784–3788. doi: 10.1093/nar/gkg563, PMID: 12824418 PMC168970

[ref18] Haist K. Ziegler C. Botten J. (2015). Strand-specific quantitative reverse transcription-polymerase chain reaction assay for measurement of Arenavirus genomic and Antigenomic RNAs. PLoS One 10:e0120043. doi: 10.1371/journal.pone.0120043, PMID: 25978311 PMC4433285

[ref19] Heinig M. Frishman D. (2004). STRIDE: a web server for secondary structure assignment from known atomic coordinates of proteins. Nucleic Acids Res. 32, W500–W502. doi: 10.1093/nar/gkh429, PMID: 15215436 PMC441567

[ref20] Holm L. Kääriäinen S. Rosenström P. Schenkel A. (2008). Searching protein structure databases with DaliLite v.3. Bioinformatics 24, 2780–2781. doi: 10.1093/bioinformatics/btn507, PMID: 18818215 PMC2639270

[ref21] Holm L. Rosenström P. (2010). Dali server: conservation mapping in 3D. Nucleic Acids Res. 38, W545–W549. doi: 10.1093/nar/gkq366, PMID: 20457744 PMC2896194

[ref22] Hoover W. G. (1985). Canonical dynamics: equilibrium phase-space distributions. Phys. Rev. A 31, 1695–1697. doi: 10.1103/PhysRevA.31.1695, PMID: 9895674

[ref23] Humphrey W. Dalke A. Schulten K. (1996). VMD: visual molecular dynamics. J. Mol. Graph. 14, 33–38. doi: 10.1016/0263-7855(96)00018-5 8744570

[ref24] Jones D. T. Cozzetto D. (2015). DISOPRED3: precise disordered region predictions with annotated protein-binding activity. Bioinformatics 31, 857–863. doi: 10.1093/bioinformatics/btu744, PMID: 25391399 PMC4380029

[ref25] Jumper J. Evans R. Pritzel A. Green T. Figurnov M. Ronneberger O.. (2021). Highly accurate protein structure prediction with AlphaFold. Nature 596, 583–589. doi: 10.1038/s41586-021-03819-2, PMID: 34265844 PMC8371605

[ref26] Kräutler V. van Gunsteren W. F. Hünenberger P. H. (2001). A fast SHAKE algorithm to solve distance constraint equations for small molecules in molecular dynamics simulations. J. Comput. Chem. 22, 501–508. doi: 10.1002/1096-987X(20010415)22:5<501::AID-JCC1021>3.0.CO;2-V

[ref27] Laverdure S. Gross A. Arpin-Andre C. Clerc I. Beaumelle B. Barbeau B.. (2012). HIV-1 antisense transcription is preferentially activated in primary monocyte-derived cells. J. Virol. 86, 13785–13789. doi: 10.1128/jvi.01723-12, PMID: 23035216 PMC3503093

[ref28] Liu D. C. Nocedal J. (1989). On the limited memory BFGS method for large scale optimization. Math. Program. 45, 503–528. doi: 10.1007/BF01589116

[ref29] Liu Z. Torresilla C. Xiao Y. Nguyen P. T. Caté C. Barbosa K.. (2018). HIV-1 antisense protein of different clades induces autophagy and associates with the autophagy factor p62. J. Virol. 93, 1–20. doi: 10.1128/jvi.01757-18, PMID: 30404795 PMC6321906

[ref30] Ludwig L. B. Ambrus J. L. Krawczyk K. A. Sharma S. Brooks S. Hsiao C. B.. (2006). Human immunodeficiency virus-type 1 LTR DNA contains an intrinsic gene producing antisense RNA and protein products. Retrovirology 3, 1–20. doi: 10.1186/1742-4690-3-80, PMID: 17090330 PMC1654176

[ref31] Madeira F. Pearce M. Tivey A. R. N. Basutkar P. Lee J. Edbali O.. (2022). Search and sequence analysis tools services from EMBL-EBI in 2022. Nucleic Acids Res. 50, W276–W279. doi: 10.1093/nar/gkac240, PMID: 35412617 PMC9252731

[ref32] Madhavi Sastry G. Adzhigirey M. Day T. Annabhimoju R. Sherman W. (2013). Protein and ligand preparation: parameters, protocols, and influence on virtual screening enrichments. J. Comput. Aided Mol. Des. 27, 221–234. doi: 10.1007/s10822-013-9644-8, PMID: 23579614

[ref33] Martí-Renom M. A. Stuart A. C. Fiser A. Sánchez R. Melo F. Sali A. (2000). Comparative protein structure modeling of genes and genomes. Annu. Rev. Biophys. Biomol. Struct. 29, 291–325. doi: 10.1146/annurev.biophys.29.1.291 10940251

[ref34] Martyna G. J. Tobias D. J. Klein M. L. (1994). Constant pressure molecular dynamics algorithms. J. Chem. Phys. 101, 4177–4189. doi: 10.1063/1.467468

[ref35] McGuffin L. J. Bryson K. Jones D. T. (2000). The PSIPRED protein structure prediction server. Bioinformatics 16, 404–405. doi: 10.1093/bioinformatics/16.4.404 10869041

[ref36] Michael N. L. Vahey M. T. d’Arcy L. Ehrenberg P. K. Mosca J. D. Rappaport J.. (1994). Negative-strand RNA transcripts are produced in human immunodeficiency virus type 1-infected cells and patients by a novel promoter downregulated by tat. J. Virol. 68, 979–987. doi: 10.1128/jvi.68.2.979-987.1994, PMID: 8289399 PMC236536

[ref37] Miller R. H. (1988). Human immunodeficiency virus may encode a novel proten on the genomic DNA plus strand. Science 239, 1420–1422. doi: 10.1126/science.3347840, PMID: 3347840

[ref38] Nordentoft I. Jørgensen P. (2003). The acetyltransferase 60 kDa trans-acting regulatory protein of HIV type 1-interacting protein (Tip60) interacts with the translocation E26 transforming-specific leukaemia gene (TEL) and functions as a transcriptional co-repressor. Biochem. J. 374, 165–173. doi: 10.1042/BJ20030087, PMID: 12737628 PMC1223570

[ref39] Nosé S. (1984). A molecular dynamics method for simulations in the canonical ensemble. Mol. Phys. 52, 255–268. doi: 10.1080/00268978400101201

[ref41] Pavesi A. Vianelli A. Chirico N. Bao Y. Blinkova O. Belshaw R.. (2018). Overlapping genes and the proteins they encode differ significantly in their sequence composition from non-overlapping genes. PLoS One 13:e0202513. doi: 10.1371/journal.pone.0202513, PMID: 30339683 PMC6195259

[ref42] Pelchen-Matthews A. Giese S. Mlčochová P. Turner J. Marsh M. (2012). β2 integrin adhesion complexes maintain the integrity of HIV-1 assembly compartments in primary macrophages. Traffic 13, 273–291. doi: 10.1111/j.1600-0854.2011.01306.x, PMID: 22017400

[ref43] Roy A. Kucukural A. Zhang Y. (2010). I-TASSER: a unified platform for automated protein structure and function prediction. Nat. Protoc. 5, 725–738. doi: 10.1038/nprot.2010.5, PMID: 20360767 PMC2849174

[ref44] Savoret J. Chazal N. Moles J.-P. Tuaillon E. Boufassa F. Meyer L.. (2020). A pilot study of the humoral response against the AntiSense protein (ASP) in HIV-1-infected patients. Front. Microbiol. 11:20. doi: 10.3389/fmicb.2020.00020, PMID: 32117090 PMC7025555

[ref45] Schrödinger Release 2022-3: Maestro (2021). New York, NY: Schrödinger, LLC. Available at: https://www.schrodinger.com/products/maestro

[ref46] Shen M. Sali A. (2006). Statistical potential for assessment and prediction of protein structures. Protein Sci. 15, 2507–2524. doi: 10.1110/ps.062416606, PMID: 17075131 PMC2242414

[ref47] Shivakumar D. Harder E. Damm W. Friesner R. A. Sherman W. (2012). Improving the prediction of absolute solvation free energies using the next generation OPLS force field. J. Chem. Theory Comput. 8, 2553–2558. doi: 10.1021/ct300203w, PMID: 26592101

[ref48] Stadelmayer B. Micas G. Gamot A. Martin P. Malirat N. Koval S.. (2014). Integrator complex regulates NELF-mediated RNA polymerase II pause/release and processivity at coding genes. Nat. Commun. 5:5531. doi: 10.1038/ncomms6531, PMID: 25410209 PMC4263189

[ref49] Torresilla C. Larocque E. Landry S. Halin M. Coulombe Y. Masson J.-Y.. (2013). Detection of the HIV-1 minus-Strand-encoded antisense protein and its association with autophagy. J. Virol. 87, 5089–5105. doi: 10.1128/jvi.00225-13, PMID: 23427159 PMC3624327

[ref50] Vanhée-Brossollet C. Thoreau H. Serpente N. D’Auriol L. Lévy J. P. Vaquero C. (1995). A natural antisense RNA derived from the HIV-1 env gene encodes a protein which is recognized by circulating antibodies of HIV+ individuals. Virology 206, 196–202. doi: 10.1016/s0042-6822(95)80034-4, PMID: 7831774

[ref51] Xu D. Zhang Y. (2011). Improving the physical realism and structural accuracy of protein models by a two-step atomic-level energy minimization. Biophys. J. 101, 2525–2534. doi: 10.1016/j.bpj.2011.10.024, PMID: 22098752 PMC3218324

[ref52] Yang J. Yan R. Roy A. Xu D. Poisson J. Zhang Y. (2015). The I-TASSER suite: protein structure and function prediction. Nat. Methods 12, 7–8. doi: 10.1038/nmeth.3213, PMID: 25549265 PMC4428668

[ref53] Zapata J. C. Campilongo F. Barclay R. A. DeMarino C. Iglesias-Ussel M. D. Kashanchi F.. (2017). The human immunodeficiency virus 1 ASP RNA promotes viral latency by recruiting the Polycomb repressor complex 2 and promoting nucleosome assembly. Virology 506, 34–44. doi: 10.1016/j.virol.2017.03.002, PMID: 28340355 PMC5505171

[ref54] Zhang Y. (2008). I-TASSER server for protein 3D structure prediction. BMC Bioinformatics 9:40. doi: 10.1186/1471-2105-9-40, PMID: 18215316 PMC2245901

