# Supplementary material for: Structural analysis and molecular dynamics simulation studies of HIV-1 antisense protein predict its potential role in HIV replication and pathogenesis
Source: Front Microbiol. 2023 Mar 20;14:1152206. doi: 10.3389/fmicb.2023.1152206 (PMC10067880; doi:10.3389/fmicb.2023.1152206)
Supplement: Supplementary file 1 [file Data_Sheet_1.docx]

***Supplementary Material***

**Structural Analysis and Molecular Dynamics Simulation studies of HIV-1 Antisense Protein (ASP) predicts its potential role in HIV replication and pathogenesis**

Balakumaran Sathiyamani ^a^, Evangeline Ann Daniel^a,^ Samdani Ansar^b,^ Bennett Henzeler Esakialraj^a^, Sameer Hassan^c^, Prasanna D Revanasiddappa^d^, Amrutha Keshavamurthy^d^, Sujata Roy^e^, Umashankar Vetrivel^a,^ *, Luke Elizabeth Hanna^a,^ *

1. Department of Virology and Biotechnology, National Institute for Research in Tuberculosis, Chetpet, Chennai-600031, Tamil Nadu, India
2. Centre for Bioinformatics, Vision Research Foundation, Sankara Nethralaya, Chennai-600006, Tamil Nadu, India
3. Department of Biosciences and Nutrition, Karolinska Institutet, Huddinge-171 77, Sweden
4. Department of Biotechnology, Siddaganga Institute of Technology, Tumakuru, Bangalore-572103, Karnataka, India
5. Department of Biotechnology, Rajalakshmi Engineering College, Chennai-602105, Tamil Nadu, India

***** Correspondence: Luke Elizabeth Hanna; Umashankar Vetrivel.

**Email:**  [hannatrc@yahoo.com](mailto:hannatrc@yahoo.com); [vumashankar@gmail.com](mailto:vumashankar@gmail.com)

Address: Department of Virology and Biotechnology, National Institute for Research in Tuberculosis, Chetpet, Chennai-600031, Tamil Nadu, India


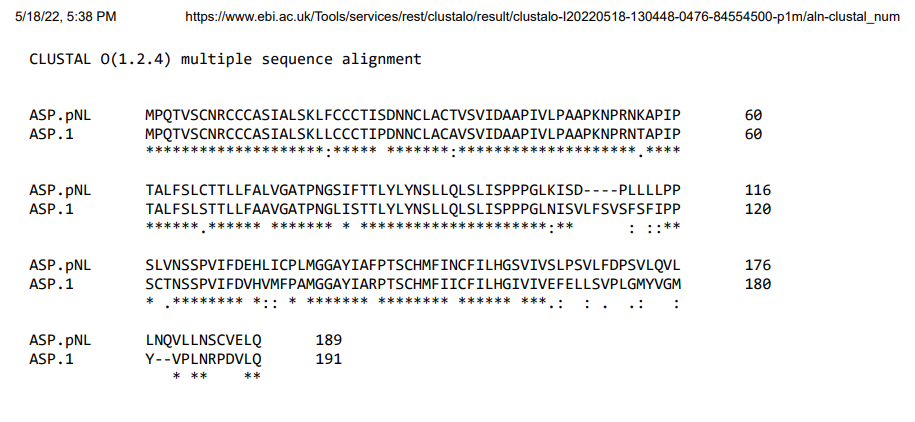


**Supplementary Figure 1**. Multiple Sequence Alignment of ASP_Indie-C1 and ASP_NL4-3 using CLUSTAL-O


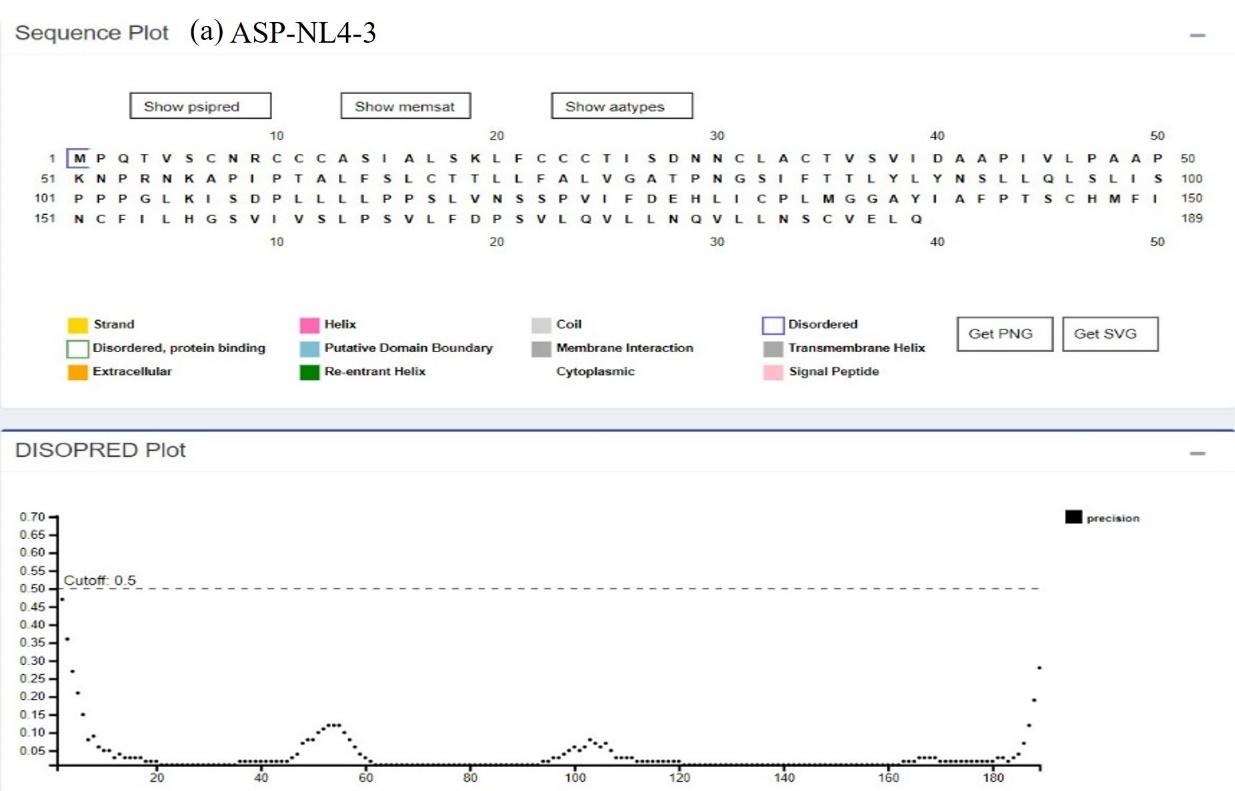


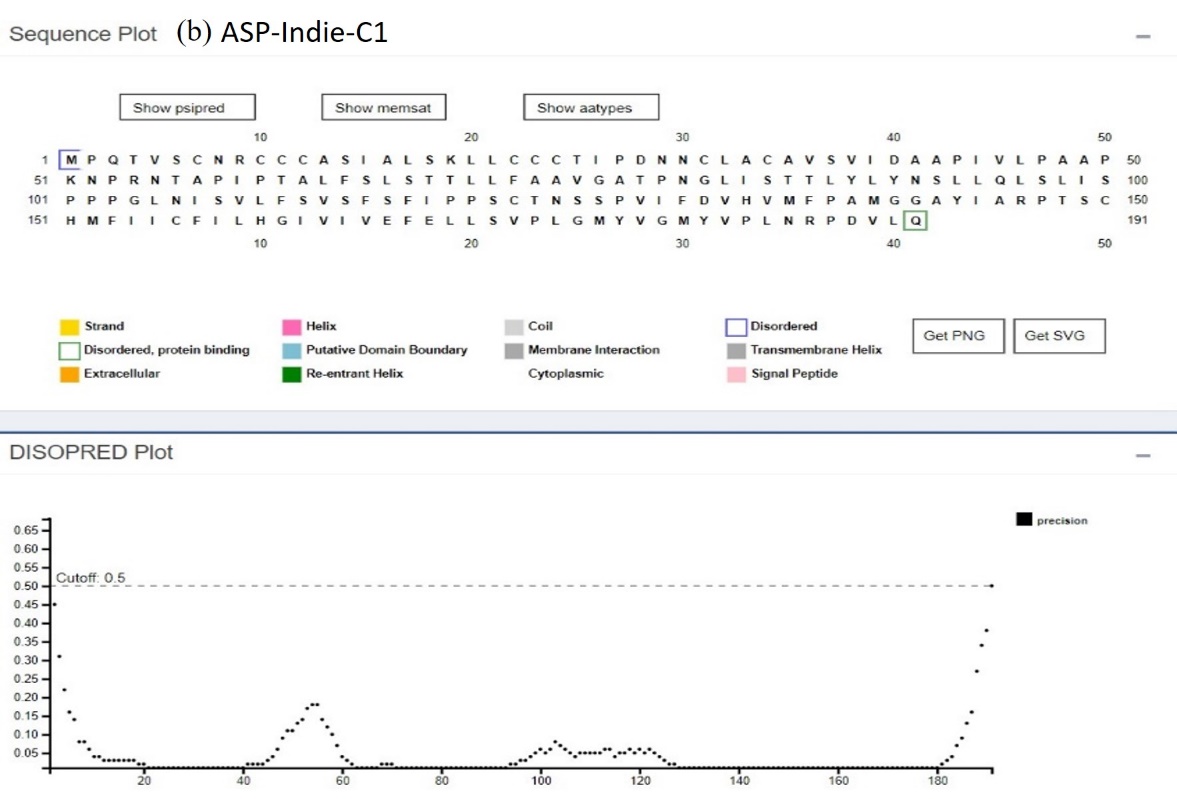


**Supplementary Figure 2**. Structural disorder prediction reveals that all the residues are reside within the cut-off value (a) ASP-NL4-3 (b) ASP-Indie-C1


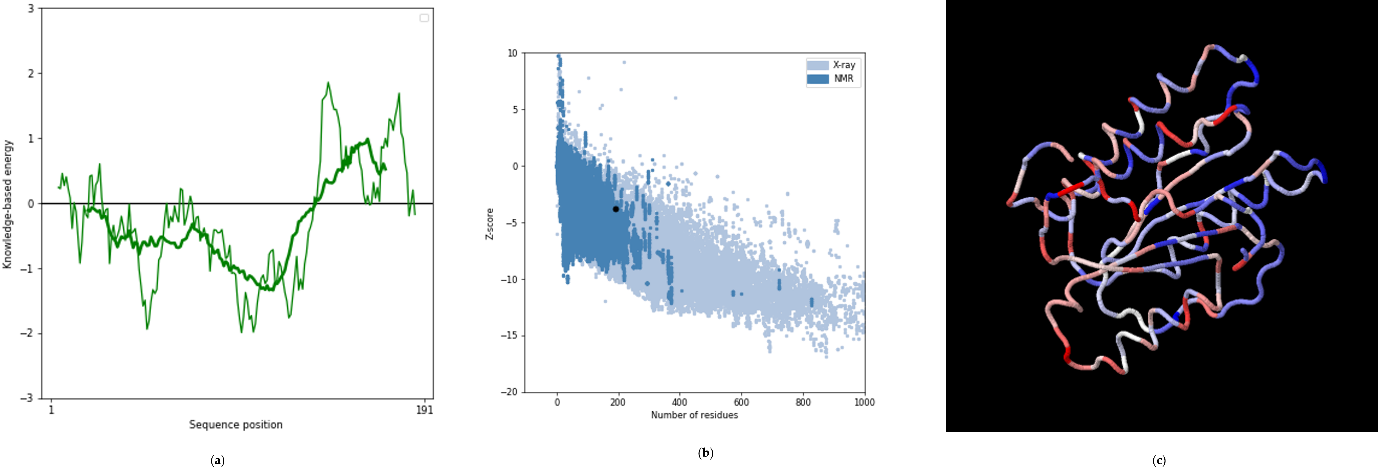


**Supplementary Figure 3**. PROSA scores of ASP_Indie-C1 (**a**) Local model quality (**b**) Overall model quality (**c**) energy scores


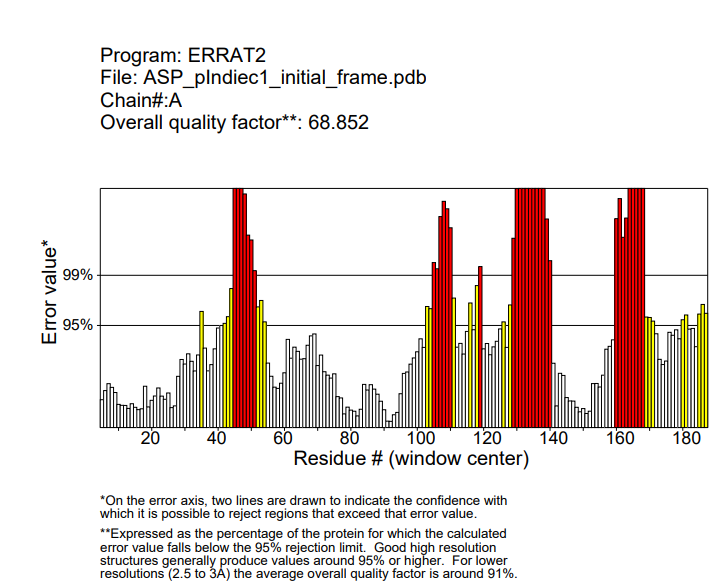


**Supplementary Figure 4**. ERRAT score of ASP_Indie_C1


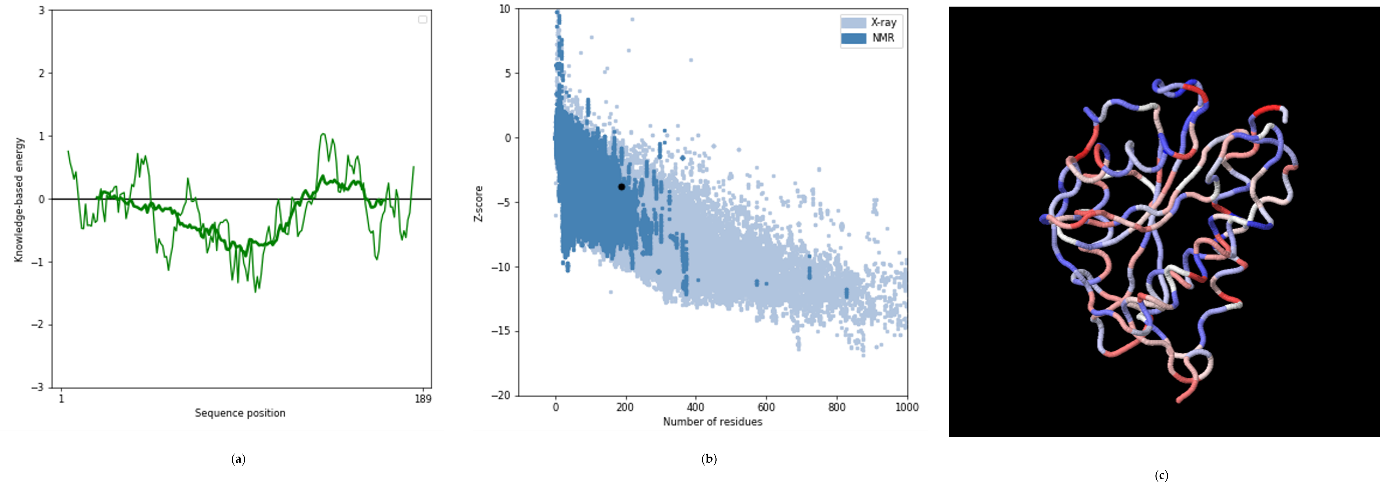


**Supplementary Figure 5**. PROSA scores of ASP_NL4-3 (**a**) Local model quality (**b**) Overall model quality (**c**) energy scores


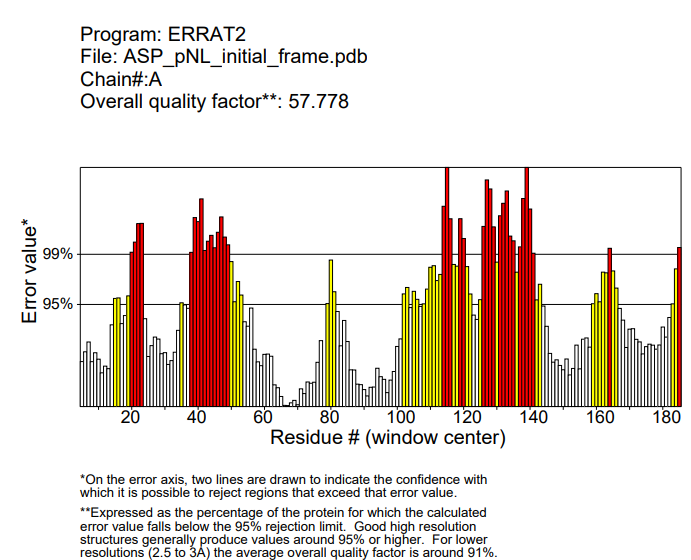


**Supplementary Figure 6**. ERRAT score of ASP_NL4-3

**
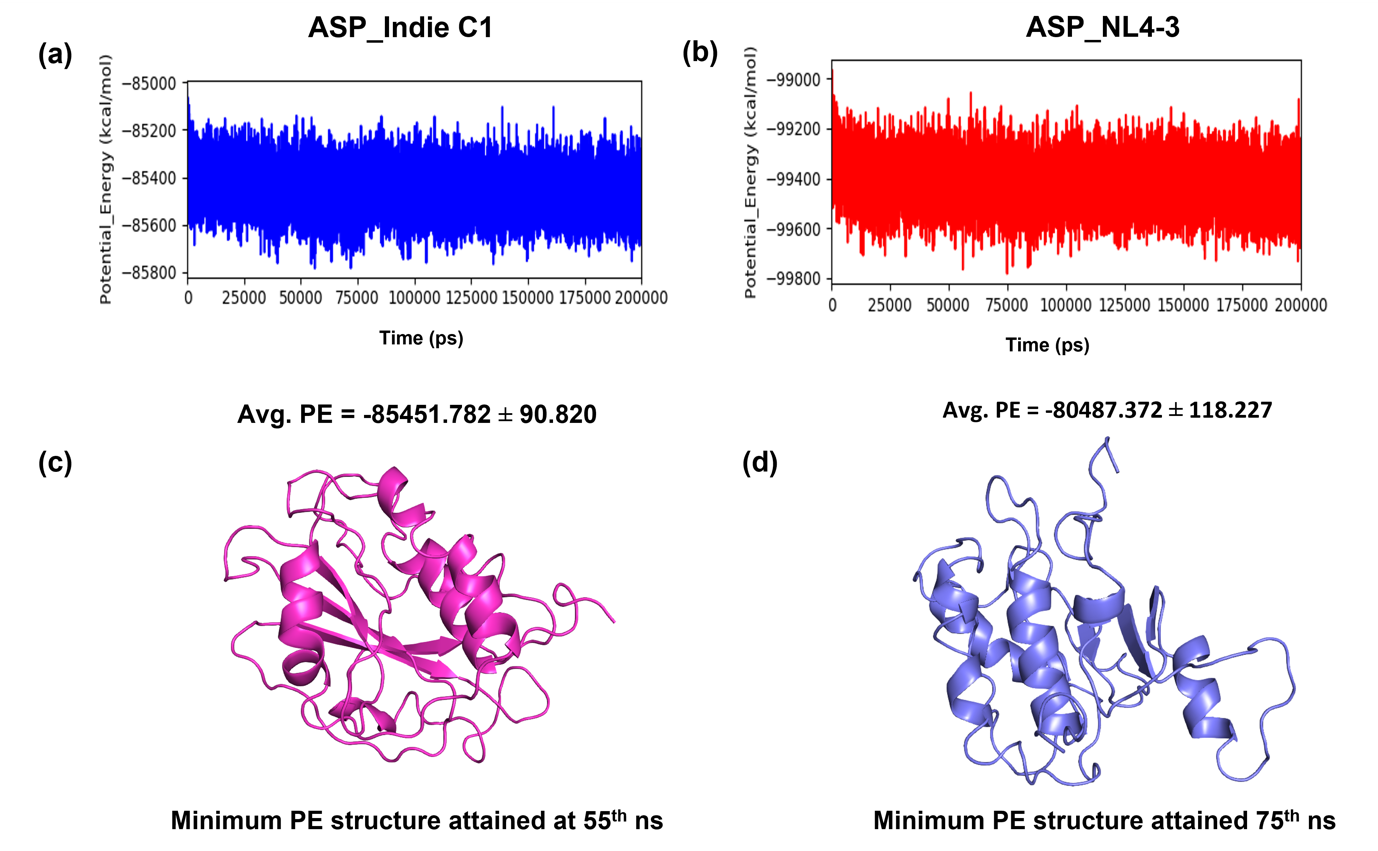
Supplementary Figure 7.** Potential energies of (a) ASP_Indie-C1 and (b) ASP_NL4-3 throughout the 200ns simulation. Conformational structure at the minimum potential energy of (c) ASP_Indie-C1 and (d) ASP_NL4-3 during the simulation.


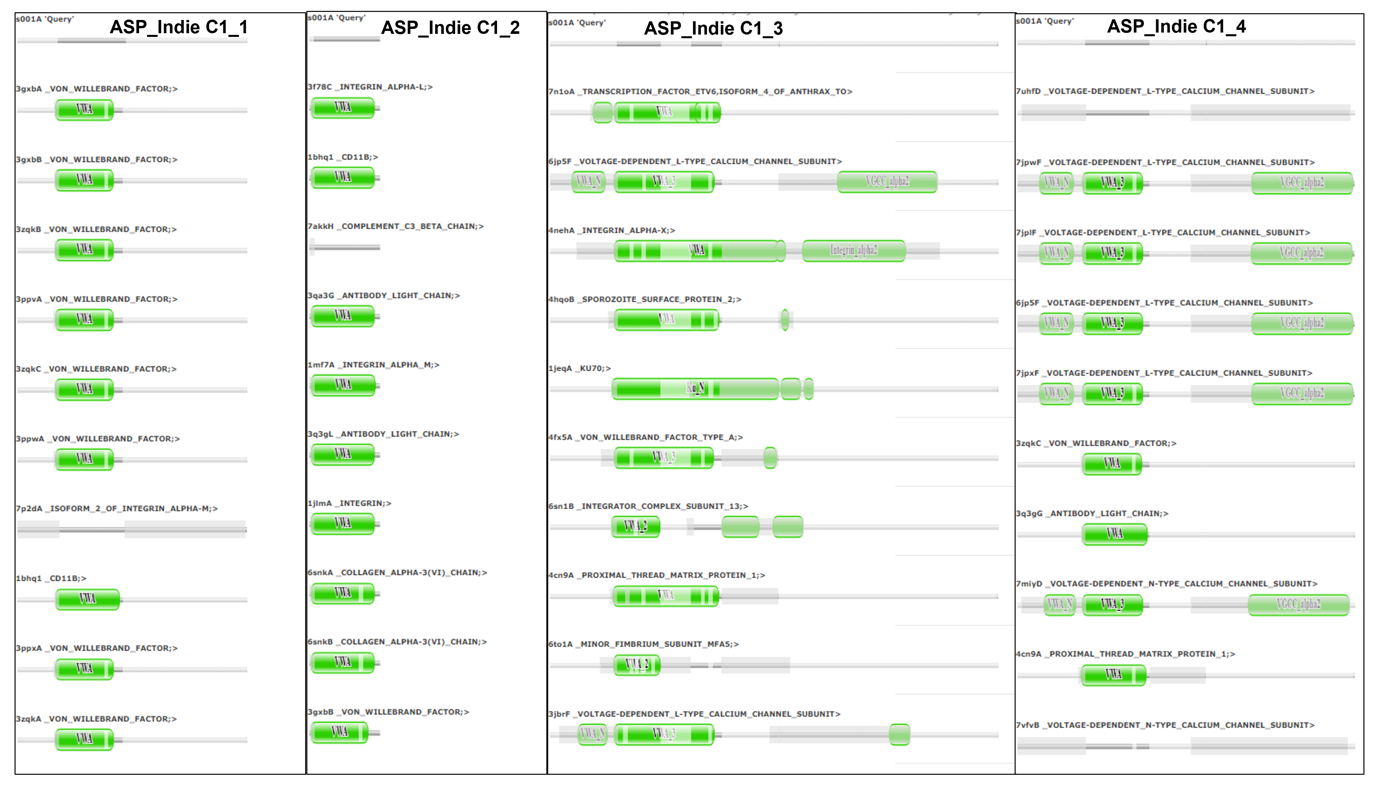


**Supplementary Figure 8.** Hits obtained from DALI server by submitting representative frame structure from each centroid cluster of ASP_Indie-C1


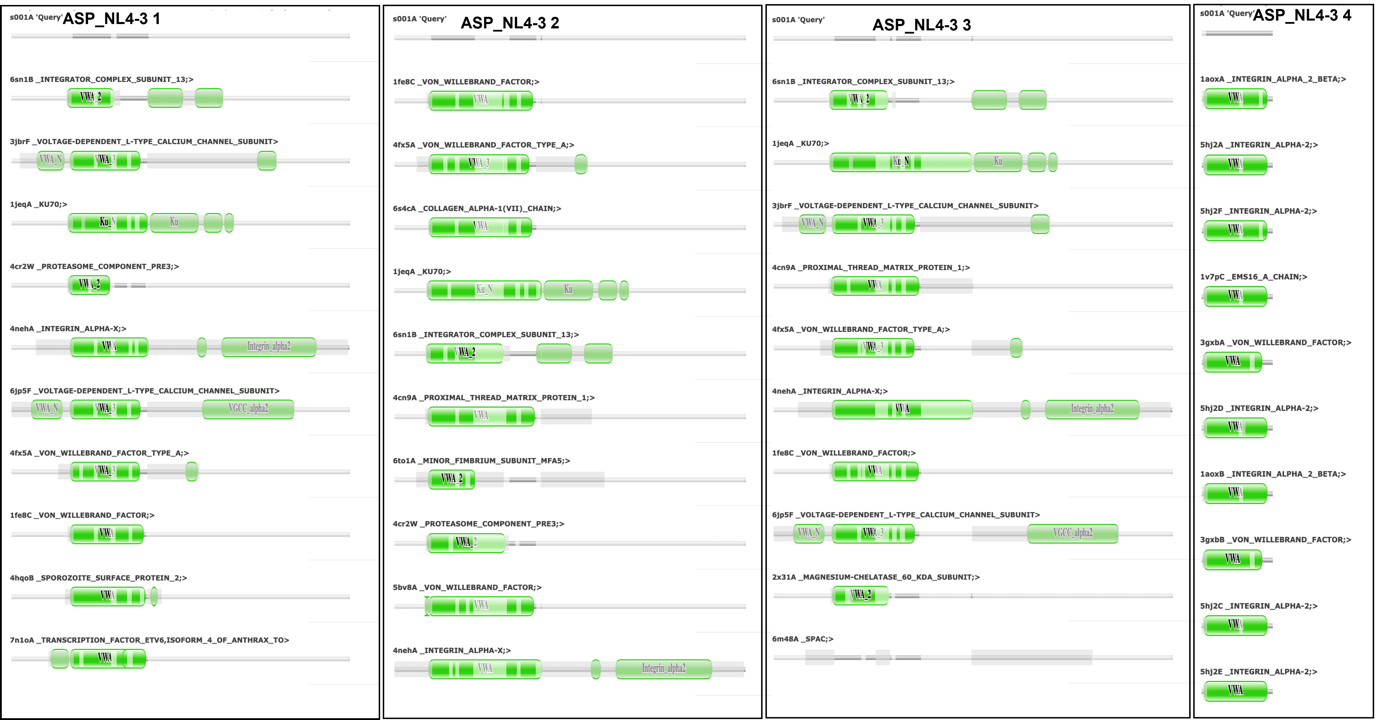


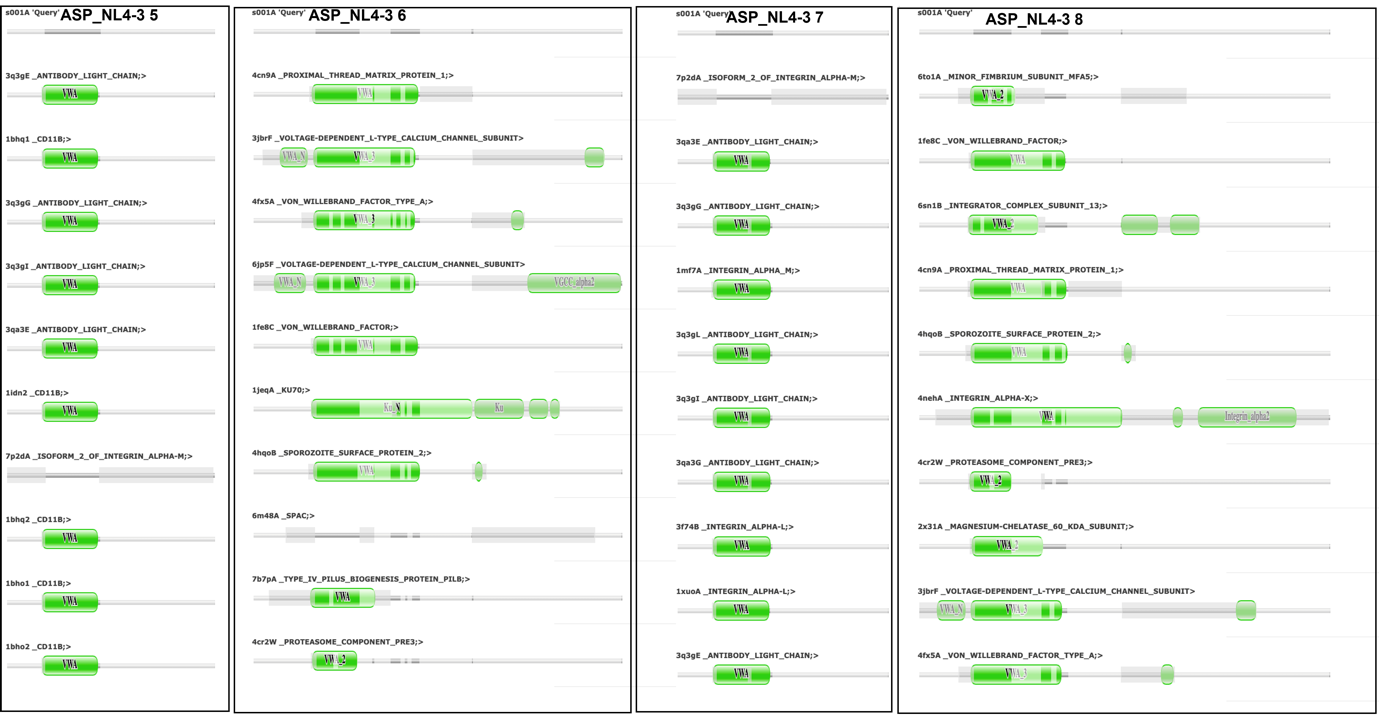


**Supplementary Figure 9.** Hits obtained from DALI server by submitting representative frame structure from each centroid cluster of ASP_NL4-3.


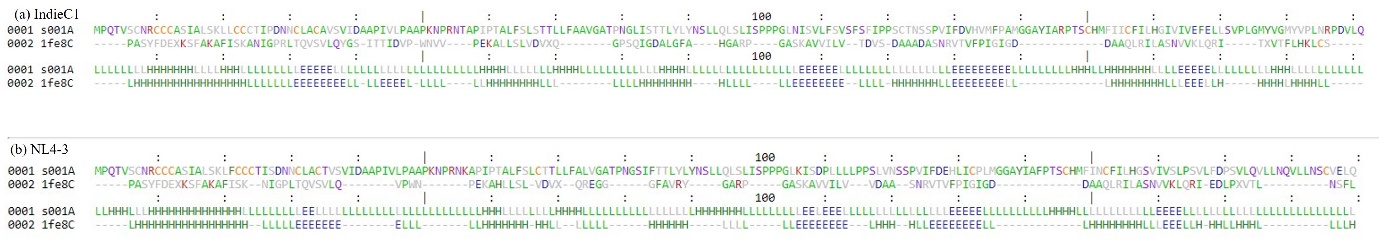


**Supplementary Figure 10.** (a) Structural alignment of Indie-C1 with VWA domain

(b) Structural alignment of NL4-3 with VWA domain
